# Efficacy of stem cells versus microvesicles in ameliorating chronic renal injury in rats (histological and biochemical study)

**DOI:** 10.1038/s41598-024-66299-0

**Published:** 2024-07-18

**Authors:** Maha Z. Mohammed, Shaimaa A. Abdelrahman, Amal S. El-Shal, Abeer A. Abdelrahman, Marwa Hamdy, Walaa M. Sarhan

**Affiliations:** 1 Medical Histology & Cell Biology Department, Faculty of Medicine, Zagazig University, Zagazig, Egypt; 2 Medical Biochemistry and Molecular Biology Department, Faculty of Medicine, Zagazig University, Zagazig, Egypt; 3 Medical Biochemistry and Molecular Biology Department, Armed Forces College of Medicine (AFCM), Cairo, Egypt; 4 Medical Biochemistry and Molecular Biology Department, Faculty of Medicine, Ain Shams University, Cairo, Egypt

**Keywords:** Stem cells, Microvesicles, Chronic renal injury, Rats, Biochemistry, Cell biology, Molecular biology, Stem cells, Medical research, Nephrology

## Abstract

Chronic exposure to heavy metals as aluminum chloride (AlCl_3_) could result in severe health hazards such as chronic renal injury. The present study aimed to evaluate the therapeutic potential of adipose tissue-derived stem cells (ASCs) in comparison to their microvesicles (MV) in AlCl_3_-induced chronic renal injury. Forty-eight adult male Wistar rats were divided into four groups: Control group, AlCl_3_-treated group, AlCl_3_/ASC-treated group, and AlCl_3_/MV-treated group. Biochemical studies included estimation of serum urea and creatinine levels, oxidative biomarkers assay, antioxidant biomarkers, serum cytokines (IL-1β, IL-8, IL-10, and IL-33), real time-PCR analysis of renal tissue MALT1, TNF-α, IL-6, and serum miR-150-5p expression levels. Histopathological studies included light and electron microscopes examination of renal tissue, Mallory trichrome stain for fibrosis, Periodic acid Schiff (PAS) stain for histochemical detection of carbohydrates, and immunohistochemical detection of Caspase-3 as apoptosis marker, IL-1B as a proinflammatory cytokine and CD40 as a marker of MVs. AlCl_3_ significantly deteriorated kidney function, enhanced renal MDA and TOS, and serum cytokines concentrations while decreased the antioxidant parameters (SOD, GSH, and TAC). Moreover, serum IL-10, TNF-α, miR-150-5p, and renal MALT1 expression values were significantly higher than other groups. Kidney sections showed marked histopathological damage in both renal cortex and medulla in addition to enhanced apoptosis and increased inflammatory cytokines immunoexpression than other groups. Both ASCs and MVs administration ameliorated the previous parameters levels with more improvement was detected in MVs-treated group. In conclusion: ASCs-derived MVs have a promising ameliorating effect on chronic kidney disease.

## Introduction

Humans and animals are exposed daily to chemicals and heavy metals like aluminum, mercury, lead, and cadmium. They represent the most important hazardous substances that can bioaccumulate in the body tissues with low excretion^1^. Aluminum (Al) is the third most prevalent element in the earth’s crust, comprising approximately 8% of it. The gastrointestinal and respiratory tracts are the entry points for Al into the human body. People are more at risk of Al poisoning due to environmental contamination, particularly wastewater^2,3^. Numerous food sources of Al include corn, yellow cheese, grain products (flour), salt, spices, vegetables, and tea leaves. It is also found in food additives and is added to water for purification. Additionally, it can be found in cosmetics, kitchenware, and containers^4^.

Regarding the medical industry, Al compounds are extensively employed in the formulation of several antacids, phosphate binders, toothpaste, buffered aspirins, vaccinations, injectable allergies, and antiperspirants (Aluminum chloride hexahydrate)^5^. Toxicological effects of Al include neurological diseases like Alzheimer’s disease and dialysis encephalopathy^6^, damaging impacts on both male and female reproductive systems^7,8^, hepatotoxicity^9^, and microcytic hypochromic anemia^10^.

Chronic renal injury could result from renal Al retention at higher doses as it has a relatively limited ability to leave the human body, and urine is the main route of excretion^11^. It causes injuries to renal tissue that exceed the regeneration rate, resulting in irreversible renal damage with time^12^. The development of chronic kidney disease was found to be associated with a large group of long noncoding RNAs(lncRNAs) which are 200-nt in length and act as regulatory molecules without protein-coding^13^. MicroRNAs (miRNAs) have also been implicated in the development of several diseases, including chronic and acute kidney disease, diabetic nephropathy (DN), hypertensive nephropathy, kidney immune diseases, and polycystic kidney disease (PKD)^14^. MicroRNAs like miR-150-5p, are increasingly recognized as critical players in the pathogenesis of kidney diseases^15^. In addition, Metastasis–Associated Lung Adenocarcinoma Transcript 1 (MALAT1) is one of the lncRNAs that are utilized as an early biomarker of metastasis in non–small cell lung cancer^16^. It encodes an 8.7-kb exon transcript and is expressed in different organs including the brain, kidney, and other tissues^17^. MALAT1 is critical in proliferation, apoptosis, and oxidative stress^18,19^.

From all mentioned above, searching for new strategies that could help in the treatment of AlCl_3_-induced chronic renal injury is critical, especially in populations with unavoidable chronic exposure to AlCl_3_. Regenerating or replacing damaged cells, tissues, or organs to restore their normal functions is the practice of regenerative medicine^20^. Cell therapy includes techniques that use living cells to treat illness and restore the biological functions of damaged organs or tissues^21^. Mesenchymal stem cells (MSCs) are mostly found in adult tissues including adipose tissue and bone marrow. They are also located in the umbilical cord and amniotic fluid in fetal tissues^22^. They offer a potentially effective alternate strategy for treating a variety of human ailments and can reduce inflammation and restore damaged tissue. These cells are widely distributed, not immunogenic, and can evade immune cell detection^23^. Reference^24^ stated that MSCs can develop into a wide range of adult cell types under the right circumstances. Moreover, they have a huge potential for replacing cells in tissues with a very low intrinsic renewal capacity like the heart and brain tissue because of their migration, homing, differentiation, and paracrine effects^25,26^.

Adipose tissue-derived mesenchymal stem cells (ASCs) represent a kind of adult stem cell that has great potential in multidirectional differentiation. They can be extracted from the stromal vascular fraction (SVF) of adipose tissues^27^. They can differentiate into adipocytes, chondrocytes, myocytes, osteoblasts, and neurocytes in vitro by using the right medium and signals^28^. The white adipose tissue is the primary source of ASCs, which have a stronger antiapoptotic capacity than those obtained from brown adipose tissue. One benefit of using ASCs is the straightforward access through subcutaneous aspiration, which is a less painful process than removing bone marrow stem cells. Unlike embryonic stem cells, they are obtained from autologous fat. So, there are fewer ethical debates surrounding their use^29,30^.

Mesenchymal stem cells not only function by replacing damaged tissues, but they also have paracrine actions mediated by the release of both soluble and encapsulated factors. The extracellular vesicles (EVs) released from MSCs are now being recognized as an integral component of intercellular communication^31^. Toh et al*.*^32^ stated that EVs are filled with trophic mediators by MSCs. They can transport mRNA, microRNA, mitochondrial components, and pro-regenerative substances, over vast distances. They exchange these bioactive molecules with nearby cells or spread genetic material to distant organs^33^. Extracellular vesicles (EVs) are also secreted in body fluids including saliva, blood, urine, breast milk, amniotic fluid, and cerebrospinal fluid. Depending on their size, origin, biogenesis, and composition, they are divided into exosomes, microvesicles, and apoptotic bodies. The two most significant subtypes of EVs are microvesicles (MVs) and exosomes^34^. Numerous investigations revealed that MSC-derived microvesicles (MSC-MVs), exhibit pro-angiogenic and cellular protective properties, and can be employed for tissue regeneration^35^. Consequently, they represent a viable therapeutic option for several pathological disorders such as nervous tissue pathology, liver fibrosis, kidney damage, and myocardial infarction^36^. Therefore, we aimed in the present study to investigate in depth the histopathological changes and the possible molecular mechanisms underpinning the therapeutic potential of ASCs in comparison to MVs in AlCl_3_-induced chronic renal damage in rats.

## Materials and methods

### Animals

Forty-eight adult healthy male Wistar rats (180–200 g) were kept at room temperature and light/dark (12/12 h) cycle and allowed a normal balanced diet and tap water. All experimental procedures were performed following the guidelines of the Institutional Animal Care and Use Committee (IACUC) approved by the Faculty of Medicine, Zagazig University, Zagazig, Egypt (Approval number: ZU IACUC/3/F/299/2022) and following ARRIVE guidelines.

#### Drugs and chemicals

##### AlCl_3_

It was bought from the Egyptian company Cairo Pharma Co. It was finalized at a concentration of 20 mg/kg/bwt in 0.9% saline. The dose for AlCl_3_ was chosen following earlier dose–response investigations, which revealed severe kidney damage at a comparable dose and administration duration^37,38^.

##### The primary antibodies used for immunohistochemistry

- Caspase3 Mouse Monoclonal Antibody (Catalog #43-780) and IL-1 beta Polyclonal Antibody (Catalog # PA5-105048), were obtained from Thermo Fisher Scientific, USA.
- Rabbit monoclonal Anti-CD40 antibody [EPR20735] (Catalog # ab224639) at 1/250 dilution. It was obtained from Abcam, US.

### Experimental design

Rats were placed into four groups at random.

- Group I (Control group): 18 rats were evenly divided into two subgroups: subgroup Ia; which received no treatment, and subgroup Ib; where each rat received 2 mL of 0.9% sodium chloride (NaCl) orally by gavage.
- Group II (AlCl_3_-treated group): AlCl_3_ was supplied orally by gavage to 10 rats at a dose of 20 mg/kg, dissolved in 0.9% saline, for a total of 40 days^38,39^.
- Group III (AlCl_3_/ASC-treated group): As in group II, ten rats received AlCl_3_ for 40 days. On the 41st day, each rat was given a single dose of ASCs suspended in 0.5 mL of PBS (1 × 10^7^ cells/mL) through intravenous injection into the caudal vein. Four weeks after administering the stem cells, specimens were collected^40^.
- Group IV (AlCl3/MV-treated group): As in group II, ten rats received AlCl_3_ for 40 days. On the 41st day, each rat was given a single intravenous dosage of 200 μg MVs diluted in 1 mL of PBS through the caudal vein. Samples were collected 21 days following the administration of MV^41^.

### Isolation and culture of adipose-derived mesenchymal stem cells (ASCs)

ASCs were collected from abdominal and inguinal subcutaneous fat. The adipose tissue was placed in phosphate-buffered saline (Gibco, Invitrogen) then, it was resuspended in a solution of collagenase type II (0.075%, Sigma) in HBSS solution (2 mL per gram of tissue, at 37 °C for 60 min) for enzymatic digestion. To halt the action of collagenase, fetal bovine serum (FBS) was introduced then, the digested adipose tissue was filtered through a 100-μm filter to eliminate any debris. The filtered solution was then centrifuged at 400×*g* for 10 min to obtain a cell pellet. An erythrocyte lysis buffer was then used to remove red blood cells (RBCs). The resulting cell pellet was cultured in flasks containing Dulbecco Modified Eagle Medium (DMEM, Gibco/BRL) supplemented with 10% FBS (Gibco/BRL). After 24 h, adherent cells were removed by washing with PBS. The attached cells were subsequently cultured in a 50 cm^2^ culture flask (Falcon) with DMEM media, which consisted of 10% FBS, 1% penicillin–streptomycin (Gibco/BRL), and 1.25 mg/L amphotericin B (Gibco/BRL).

When large colonies reached approximately 80% to 90% confluence, they were washed with PBS and subjected to trypsinization using 0.25% trypsin in 1 mM EDTA (Gibco/BRL) for 5 min at 37 ℃. The collected cells were then centrifuged, re-suspended in a medium, and incubated in a 50 cm^2^ Falcon. The previous steps are known as the first passage. The cells were then cultured as previously mentioned until the third passage^42^. Finally, the ASCs (1 × 10^7^ cells/mL) were intravenously injected into the caudal vein, and dissolved in 0.5 mL of PBS.

### Identification and characterization of differentiated ASCs

The cultured ASCs were identified by their spindle shape, adherence, and positive detection of ASC surface markers CD73 and CD105. They are negative for hematopoietic markers CD14, CD34, and CD106 through real-time PCR analysis (rt-PCR). The specific primer sequences used for this analysis can be found in Table 1, as referenced from the studies by Refs.^43–46^.

**Table 1:** Rat primer sequences of studied genes.

Gene	Primer sequence
CD14	Forward 5ʹ-TCACAATTCACTGCGGGATA-3ʹ Reverse 5ʹ-CGATGTCCTAGGAGCAAAGC-3ʹ
CD34	Forward 5ʹ-AGCCATGTGCTCACACATCA-3ʹ Reverse 5ʹ-CAAACACTCGGGCCTAACC-3ʹ
CD73	Forward 5ʹ-TGCATCGATATGGCCAGTCC-3ʹ Reverse 5ʹ-AATCCATCCCCACCGTTGAC-3ʹ
CD105	Forward 5ʹ-ACTGAGTTGCACATCTGGGG-3ʹ Reverse 5ʹ-TTCCGAAGTGGTGGTAAGCC-3ʹ
CD106	Forward 5ʹ-GGTGGCTGCACAGGTTGGGG-3ʹ Reverse 5ʹ-ACCCACAGGGCTCAGCGTCA-3ʹ
MALT1	Forward 5ʹ-CTTAAG CGCAGCGCCATTTT-3ʹ Reverse 5ʹ-CCTCCAAACCCCAAGACCAA-3ʹ
18s	Forward 5ʹ-CGAAAGCATTTG CCA AGA AT-3ʹ Reverse 5ʹ-AGTCGGCATCGTTTATGG TC-3ʹ
IL-6	Forward 5ʹ-GCCCTTCAGGAACAGCTATGA-3ʹ Reverse 5ʹ-CAACATCAGTCCCAAGA-3ʹ
MiR-150-5p	Forward 5ʹ-TCTCCCAACCCTTGTACCAGTG-3ʹ Reverse 5ʹ-TGGTGTCGTGGAGTCG-3ʹ
TNF-α	Forward 5ʹ-GTCGTAGCAAACCACCAAGC-3ʹ Reverse 5ʹ-TGTGGGTGAGGAGCACATAG-3ʹ
GAPDH	Forward 5ʹ-ATCCCATCACCATCTTCCAG-3ʹ Reverse 5ʹ-CACACCCATGACGAACATGGG-3ʹ
U6	Forward 5ʹ-CTCGCTTCGGCAGCACA-3ʹ Reverse 5ʹ-AACGCTTCACGAATTTGCGT-3ʹ

Following the manufacturer’s instructions, total RNA was extracted from cell culture using the RNA-spin™ total RNA extraction Kit (iNtRON Biotechnology, Korea). Subsequently, cDNA was synthesized with the cDNA synthesis Kit from iNtRON Biotechnology. For the real-time PCR reactions, a mixture was prepared with a total volume of 20 µL, consisting of 5 µL cDNA, 0.5 µL of each primer at 100 pmol/µL concentration (Biolegio), 10 µL of Eva Green PCR Master mix (Jena Bioscience), and 4 µl of PCR-grade water.

### Isolation of microvesicles

Microvesicles (MVs) were harvested from the medium of proliferating ASCs through the process of supernatant centrifugation and multiple filtrations to collect the microvesicles. The ASCs were grown in DMEM medium for 24–48 h then they were subjected to centrifugation at 123×*g* (1000 rpm) for 5 min, followed by 769×*g* (2500) rpm for 15 min, and then passed through a 0.45 µm filter to eliminate any cell debris. The conditioned medium was then ultracentrifuged using a Thermo Scientific ultracentrifuge at 100,000×*g* for 1 h at 4 °C to sediment the MVs. The resulting pellets were resuspended in PBS and stored for later use.

### Identification and characterization of MVs

Transmission electron microscopy (TEM) was used for the identification and characterization of MVs in addition to the detection of positive CD63 (extracellular MVs surface marker) by flow cytometry^47^.

### Labeling of ASCs and MVs with cell linker (PKH-26)

Both ASCs and MVs were labeled with a fluorescence marker [Paul Karl Horan (PKH26); red Fluorescent Cell Linker Kit, Sigma, St. Louis, Missouri, USA] before injection in rats according to Refs.^48,49^ respectively. A fluorescent microscope (Olympus BX50F4, No. 7M03285, Tokyo, Japan) was used for the examination of renal tissue sections of treated groups at the Biochemistry and Molecular Biology Department, Faculty of Medicine, Zagazig University, Zagazig, Egypt.

### Blood collection and tissue preparation

Rats were anesthetized using 60 mg/kg of pentobarbital administered intraperitoneally. Blood samples were obtained from the ophthalmic veins and centrifuged at 492×*g* (2000 rpm) for 20 min to obtain serum samples, which were then stored at − 20 °C for later biochemical assays. The renal tissue from each animal was split into two sections: one for histological examination and the other for biochemical and molecular studies. Tissue samples were homogenized in 5–10 mL of cold buffer (50 mM potassium phosphate, pH 7.4, 1 mM EDTA; Catalog Number G8789) per gram of tissue. This was followed by centrifugation of the homogenate at 1698×*g* (4000 rpm) for 15 min at 4 °C. The supernatant collected was then stored at − 80 °C for analysis of oxidative stress markers.

### Estimation of serum urea and creatinine levels

Serum urea and creatinine levels were determined using colorimetric methods provided by Diamond Diagnostics, Egypt, following the instructions included with the kits.

### Oxidative biomarkers assay

Malondialdehyde (MDA), an indicator of lipid peroxidation, was measured calorimetrically in renal homogenates using a kit from Biodiagnostic, Egypt (Catalog No: MD 25 29). This method is based on the reaction between thiobarbituric acid (TBA) and MDA in an acidic environment at 95 °C for 30 min, resulting in a pink TBA-MDA complex that is measurable calorimetrically at 534 nm, as described by Ohkawa et al*.*^50^. Additionally, levels of Reactive Oxygen Species (ROS) were quantified using an ELISA kit (Catalog No: MBS2802061, My BioSource, USA) following the manufacturer’s protocol.

### Measurement of antioxidant biomarkers

The total oxidative status (TOS) in renal homogenates was measured using a calorimetric method with the Rel Assay Diagnostics kit from Turkey. The findings were reported in terms of mol H_2_O_2_ equivalents per liter, calibrated against hydrogen peroxide (H_2_O_2_). The activity of superoxide dismutase (SOD) in renal tissue was determined in units per gram (U/g) using the method developed by Nishikimi et al*.*^51^. Reduced glutathione (GSH) levels were assessed by employing Beutler’s Technique^52^. While the activity of glutathione reductase (GR) was measured following the protocol by Erden and Bor^53^. The serum’s total antioxidant capacity (TAC) was evaluated based on the procedure outlined by Koracevic et al*.*^54^. All the kits for these tests were supplied from Bio Diagnostics, Cairo, Egypt (Catalog Numbers: SD 25 21, GR 25 11, GR 25 23, and TA 25 13, respectively).

### Measurement of serum cytokines concentrations

ELISA kits (My BioSource, USA) were utilized to quantify the concentrations of interleukin-1 beta (IL-1β), interleukin-8 (IL-8), interleukin-10 (IL-10), and interleukin-33 (IL-33), following the manufacturer’s guidelines (Catalog Numbers: MBS650780, MBS2019367, MBS2020828, and MBS703657, respectively).

### RNA extraction and real time-PCR analysis of MALT1& TNF-α & IL-6

The RNA-spin™ Extraction Kit (iNtRON Biotechnology, Korea) was utilized to extract total RNA from renal tissue, following the protocol provided by the manufacturer. Subsequently, RNA was reverse transcribed using a cDNA synthesis Kit from iNtRON Biotechnology, as per the manufacturer’s guidelines. For the Real-time PCR (RT-PCR) assays, a reaction mix of 20 µL was prepared, containing 5 µL of cDNA, 0.5 µL of each primer at a concentration of 100 pmol/µL (Biolegio), 10 µL of EvaGreen PCR Master mix (Jena Bioscience), and 4 µL of PCR-grade water. The primer sequences for MALT1, TNF-α, and IL-6 were detailed in Table 1^55–57^. The RT-PCR amplification protocol was as follows: initial denaturation at 95 °C for 2 min, followed by 40 cycles of 95 °C for 15 s, annealing at 55 °C for 45 s, and elongation at 65 °C for 45 s. For the normalization of each target gene, relative gene expression analysis was conducted using 18S and GAPDH as housekeeping genes, respectively for employing the comparative Ct method^58^.

### miRNA isolation, reverse transcription, and miR-150-5p expression

Total miRNA was isolated from serum utilizing the miRcut kit (Tiangen Biotech, China, Catalog Number: 4992860) following the manufacturer’s instructions. The miRNA was reverse-transcribed and polyadenylated into complementary DNA (cDNA) using the miRCURY LNA RT Kit (Qiagen GmbH, Germany, Catalog Number: 339340).

RT-PCR, conducted with an Applied Biosystems instrument, was employed to measure the expression levels of miR-150-5p. The primer kit was obtained from miRCURY (Qiagen GmbH, Germany). Amplification was carried out in a final volume of 20 µL, comprising 2 µL of 10× miScript universal reverse primer, 10 µL of 2× Quanti-Tect cyber-Green PCR master mix, and 2 µL of template cDNA, along with 2 μL of 10 pmol forward specific stem-loop primer, and 4 μL of RNase-free water. RT-PCR conditions were as follows: initial activation at 95 °C for 15 min (one cycle), denaturation at 94 °C for 40 cycles, annealing at 55 °C for 30 s, and extension at 70 °C for 30 s; each for 25 cycles^59^. Relative gene expression of miR-150-5p was assessed using U6 as the housekeeping gene for normalization via the comparative Ct method^58^.

### Histological studies

#### Light microscopic studies^60^

Kidney tissue sections from all groups were processed for microscopic examination. Parts of renal tissue underwent fixation with 10% formol saline for 24 h, after which they were processed to produce paraffin blocks. 5 μm thick Paraffin sections were cut, mounted, and stained by:

- Hematoxylin and Eosin (H&E) stains.
- Mallory trichrome stain.
- Periodic acid Schiff (PAS) staining.

#### Immunohistochemical staining^61^

Paraffin slices were deparaffinized in xylene, rehydrated, and then washed in PBS. After inactivating the endogenous peroxidase activity with 3% H_2_O_2_, sections were then washed with PBS. Then, the primary antibodies were used. The primary antibodies were identified by biotinylated secondary antibody (Zymed Laboratories; South San Francisco, CA, USA) incubation for 30 min at room temperature. After numerous PBS washes, the streptavidin–biotin peroxidase complex was then incubated with all the sections for a further 30 min at room temperature. Reactions were seen using 3,3′ Diaminobenzidine Tetrahydrochloride (DAB; Sigma-Aldrich Chemical Co., St. Louis, USA) after washing with PBS. It was used as a chromogen. Mayer’s hematoxylin was used as a counterstain. The primary antibody in the negative controls was replaced by PBS. Brown staining indicates a successful detection of the antibody. Caspase-3 was used as a cell apoptosis marker, IL-1B was used as a proinflammatory cytokine and CD40 was used as a marker for mesenchymal stem cells-derived microvesicles.

### Electron microscopic study

Kidney tissue specimens were divided into small pieces (0.5–1.0 mm^3^), prefixed in 2.5% glutaraldehyde for 2 h, and post-fixed in 1% osmium tetroxide in 0.1 M phosphate buffer (pH 7.4) at 4 °C for 2 h. Dehydration and resin embedding were done. Semithin (1 μm) and ultrathin (60–90 nm) sections were cut using a Leica ultracut (UCT) (Glienicker, Berlin, Germany) at the Electron Microscope Research Unit, Faculty of Agriculture, Mansoura University. Transmission electron microscopy (TEM, JEOLJEM-1400, Japan) was used to analyze the ultrathin slices after they had been stained with lead citrate and uranyl acetate^62^.

#### Morphometric study

The area percents (area %) of Caspase-3, IL-1B, and CD40 immune expression as well as that of PAS and Mallory trichrome stain were performed at the image analysis unit of Medical Histology and Cell Biology department, Faculty of Medicine, Zagazig University. Using Fiji image J (1.51n, NIH, USA) program by the image analyzer computer system Leica Qwin 500 (Leica Ltd, Cambridge, UK), the interactive measure menu was used to measure them. Ten readings from five distinct areas from each rat in all groups were evaluated.

#### Statistical analysis of data

It was done using Statistical Package for Social Science (SPSS), version 25.0. The least significant difference (LSD) test and one-way analysis of variance (ANOVA or F-test) were used to compare the study groups for normally distributed data. Data were expressed as mean ± SD. A *P*-value of 0.05 or less was regarded as statistically significant.

## Results

### Identification and characterization of MVs

**Figure 1:**
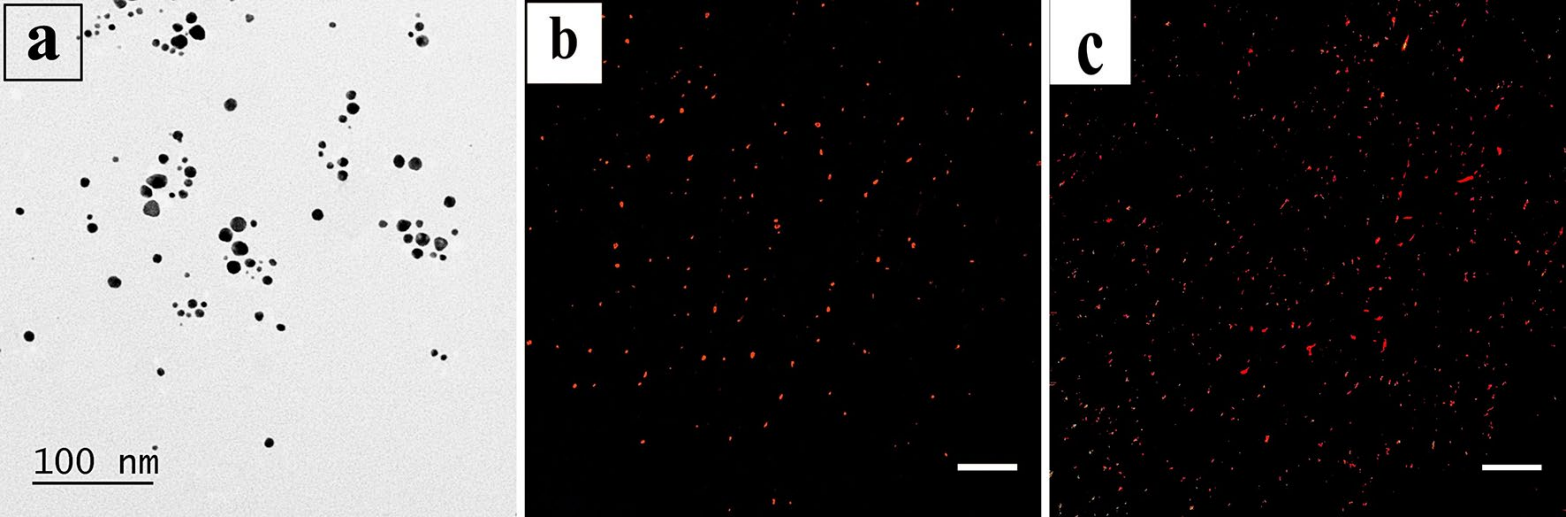
(**a**) Transmission electron microscope (TEM) examination of MVs showing spheroidal structures of heterogeneous shapes and different sizes. (**b,c**) ASCs and microvesicles-treated groups were examined by fluorescent microscope and showed PKH26 labeling that appeared as bright red dots.

Transmission electron microscope (TEM) examination of MVs showed that they appeared as spheroidal structures of heterogeneous shapes and of different sizes (Fig. 1a).

#### Detection of homing of stem cells and microvesicles

Sections in the rat kidney of ASCs and microvesicles-treated groups were examined by fluorescent microscope and showed PKH26 labeling that appeared as bright red dots (Fig. 1b,c).

### Oxidative stress biomarker concentrations

No statistically significant differences were noticed between the control subgroups regarding levels of the studied parameters. Significant variations were detected in MDA and TOS in renal homogenate between the studied groups (*P* < 0.001 for each). Group II showed significantly higher levels of renal MDA and TOS (4.90 ± 0.3 and 41.8 ± 2.6, respectively) while ASC therapy (group III) and ASC-derived MVs (Group IV) with AlCl_3_ significantly decreased their levels. On the other side, we detected significant differences in antioxidant parameters (SOD & GSH &GR) in renal homogenate, and TAC in serum between the studied groups. AlCl_3_ decreased the antioxidant parameters (SOD, GSH, and TAC) which were improved by ASC treatment. Moreover, we detected significant increases in GR concentration in group IV compared to group III (Table 2).

**Table 2:** Oxidative stress parameters in the study groups (mean ± SD).

	Group Ia	Group Ib	Group II	Group III	Group IV	P-value
MDA (nmol/g tissue)	1.73 ± 0.09	1.67 ± 0.1	4.9 ± 0.3	2.3 ± 0.1	2.3 ± 0.1	< 0.001
Post-hoc	II, III	II, III	Ia, Ib, Ic, III	Ia, Ib, II	Ia, Ib, II	
TOS umo H2O2 equivl/L	19.3 ± 1.0	20.2 ± 1.1	41.8 ± 2.6	18.7 ± 1.4	19.8 ± 1.4	< 0.001
Post-hoc	II, III	II, III	Ia, Ib, III	Ia, Ib, II	Ia, Ib, II	
SOD (U/g)	29.9 ± 2.7	30.1 ± 2.6	21.5 ± 2.0	27.3 ± 2.8	26.5 ± 2.9	0.003
Post-hoc	II, III	II, III	Ia, Ib, III	Ia, Ib, II	Ia, Ib, II	
GSH (mmol/g) tissue	9.2 ± 0.2	9.3 ± 0.4	4.1 ± 0.2	6.3 ± 0.9	7.8 ± 0.9	< 0.001
Post-hoc	II, III	II, III	Ia, Ib, III	Ia, Ib, II	Ia, Ib, II	
GR (U/g)	4.8 ± 2.9	4.3 ± 1.9	1.0 ± 1.2	3.1 ± 1.9	3.9 ± 2.5	< 0.001
Post-hoc	II, III	II, III	Ia, Ib, III	Ia, Ib, II	Ia, Ib, II,III	
TAC (mm/L)	0.78 ± 0.09	0.9 ± 0.06	0.01 ± 0.02	0.65 ± 0.06	0.69 ± 0.1	< 0.001
Post-hoc	II, III	II, III	Ia, Ib, III	Ia, Ib, II	Ia, Ib, II	

### Serum inflammatory cytokines levels in different study groups

No significant changes were detected in serum cytokines levels between both control subgroups. Significant differences in serum cytokines levels were detected between the study groups (*P* < 0.001). When comparing the AlCl_3_ group (group II) to the control group, post hoc tests revealed that serum concentrations of IL-1β and IL-8 were significantly higher, while IL-10 and IL-33 were significantly decreased. ASC therapy (group III) and ASC-derived MVs (Group IV) with AlCl_3_ improved the inflammatory status by decreasing pro-inflammatory serum cytokines (IL-1β and IL-8) and increasing anti-inflammatory serum cytokines (IL-10 and IL-33) levels suggesting potential anti-inflammatory effect (Table 3).

**Table 3:** Serum inflammatory cytokines in the study groups (mean ± SD).

	Group Ia	Group Ib	Group II	Group III	Group IV	P-value
IL-1β (pg/mL)	1.1 ± 0.18	1.14 ± 0.15	5.9 ± 0.7	3.3 ± 0.5	2.9 ± 0.4	< 0.001
Post-hoc	II, III	II, III	Ia, Ib, III	Ia, Ib, II	Ia, Ib, II	
IL-8 (pg/mL)	1.0 ± 0.5	0.9 ± 0.6	5.7 ± 2.1	3.9 ± 1.07	2.5 ± 1.07	< 0.001
Post-hoc	II, III	II, III	Ia, Ib, III	Ia, Ib, II	Ia, Ib, II	
IL-10 (pg/mL)	6.5 ± 1.4	7.1 ± 0.2	1.5 ± 0.1	3.2 ± 1.4	3.8 ± 1.2	< 0.001
Post-hoc	II, III	II, III	Ia, Ib, III	Ia, Ib, II	Ia, Ib, II	
IL-33 (pg/mL)	8.5 ± 1.4	8.2 ± 0.5	1.4 ± 0.8	5.3 ± 1.4	6.2 ± 2.1	< 0.001
Post-hoc	II, III	II, III	Ia, Ib, III	Ia, Ib, II	Ia, Ib, II	

### Serum miRNA expression in different study groups

Serum IL-10, TNF-α, miR-150-5p, and MALT1 expression values were significantly higher in group II (AlCl_3_) compared to control subgroups and ASC therapy (group III) and ASC-derived MVs (Group IV). However, we didn’t find significant changes between groups III and IV regarding miR-150-5p expression levels (Table 4).

**Table 4:** Renal expression genes in the study groups (mean ± SD).

	Group Ia	Group Ib	Group II	Group III	Group IV	P-value
IL-6	1.02 ± 0.12	1.01 ± 0.09	5.9 ± 0.8	3.3 ± 0.5	2.2 ± 0.7	< 0.001
Post-hoc	II, III	II, III	Ia, Ib, III	Ia, Ib, II	Ia, Ib, II, III	
TNF-α	1.04 ± 0.07	1.02 ± 0.05	4.9 ± 1.6	2.7 ± 0.6	1.3 ± 0.5	< 0.001
Post-hoc	II, III	II, III	Ia, Ib, III	Ia, Ib, II	Ia, Ib, II,III	
MALT1	1.05 ± 0.06	1.03 ± 0.09	6.3 ± 1.7	3.9 ± 1.3	2.8 ± 1.4	< 0.001
Post-hoc	II, III	II, III	Ia, Ib, III	Ia, Ib, II	Ia, Ib, II, III	
miR-150-5p	1.04 ± 0.08	1.09 ± 0.05	8.5 ± 1.4	6.2 ± 0.9	6.1 ± 0.7	< 0.001
Post-hoc	II, III	II, III	Ia, Ib, III	Ia, Ib, II	Ia, Ib, II	

### Renal function tests in studied groups

Serum urea and creatinine values were significantly higher in group II (AlCl_3_) compared to control subgroups and ASC therapy (group III) and ASC-derived MVs (Group IV). However, no significant changes were found between groups III and IV regarding their levels (Table 5).

**Table 5:** Renal function tests in the study groups (mean ± SD).

	Group Ia	Group Ib	Group II	Group III	Group IV	P-value
Serum urea (mg/dL)	25.3 ± 0.13	25.7 ± 0.15	67.51 ± 1.5	31.3 ± 1.15	27.66 ± 1.13	< 0.001
Serum creatinine (mg/dL)	0.56 ± 1.12	0.55 ± 1.02	2.57 ± 0.04	1.03 ± 0.04	0.63 ± 0.02	< 0.001

#### Histopathological results

**Figure 2:**
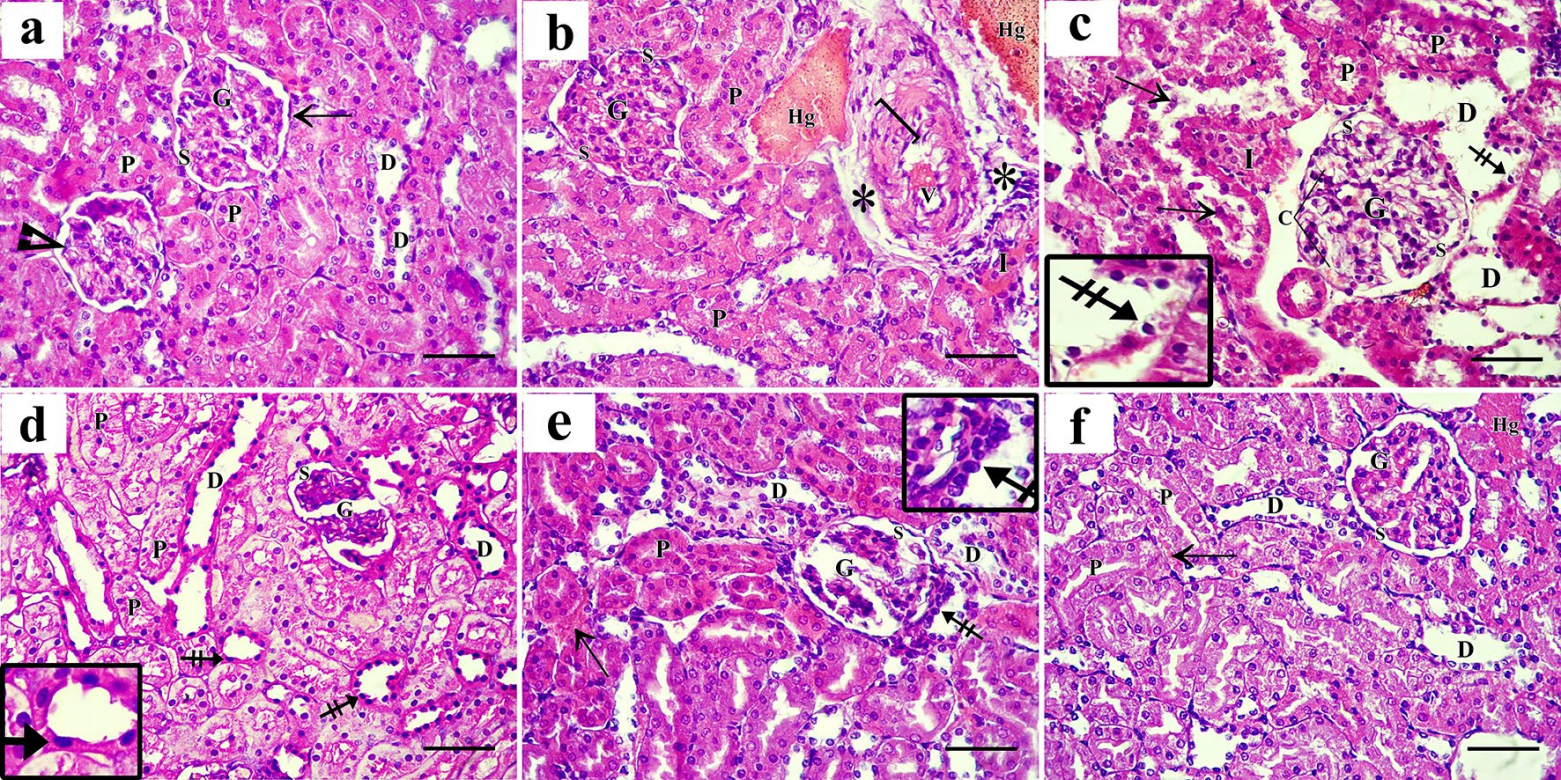
Photomicrographs of H&E-stained sections of rats’ renal cortex showing (**a**) The control group shows the glomerulus (G) is surrounded by visceral (arrowhead) and parietal (arrow) layers of Bowman’s capsule and separated by Bowman’s space (S). Many Proximal (P) and distal (D) convoluted tubules are detected. (**b**) The AlCl_3_-treated group shows congested glomerulus (G) surrounded by narrow Bowman’s space (S), Extensive interstitial hemorrhage (Hg), and inflammatory cell infiltration (I). The blood vessels are congested (v) with thick walls (]) and surrounded by wide spaces (asterisks). (**c**) Other sections of the same group show enlarged glomerulus (G) with congested glomerular capillaries (C) and Bowman’s space (S) shows unequal thickness. Desquamated cells (arrow) appear in the proximal (P) and the dilated distal (D) tubules which also show small dark nuclei (crossed arrow) and dark cytoplasm. (**d**) AlCl_3_/MSC-treated group shows atrophied glomerulus (G) with wide Bowman’s space (S), dilated distal tubules (D) lined with flattened cells having dark nuclei and cytoplasm (crossed arrow). (**e**) other sections of the same group show distorted glomerulus (G), proximal tubules with obliterated lumen and tubular cells with dark acidophilic cytoplasm (P), and residual interstitial hemorrhage (arrow). Dark crowded nuclei of Macula Densa cells are evident (crossed arrow). (**f**) AlCl_3_/MV-treated group sections show normal-shaped glomerulus (G), proximal (P), and distal tubules (D). Areas of residual hemorrhage (Hg) and congested cortical blood vessels (arrow) are observed between tubules.

**Figure 3:**
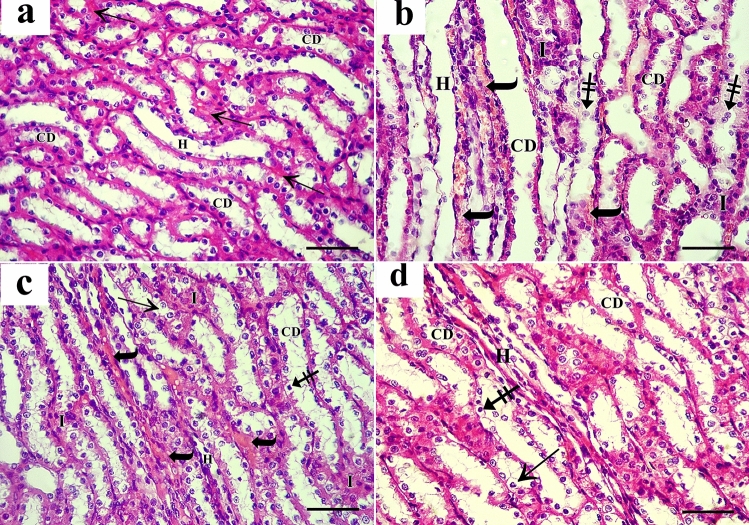
Photomicrographs of H&E-stained sections of rats’ renal medulla showing (**a**) the control group shows loops of Henle (H) lined with simple squamous epithelium, the collecting ducts (CD) lined with cuboidal cells with rounded nuclei, and vasa recta in between the duct system (arrow). (**b**) AlCl_3_-treated group shows markedly dilated loops of Henle (H) and collecting ducts (CD) with desquamated cells in the lumen (crossed arrow). The interstitial tissue shows inflammatory cell infiltration (I), and congested, dilated vasa recta (curved arrow). (**c**) AlCl_3_/MSC-treated group shows some collecting duct cells are vacuolated (arrow), and few cells have dark apoptotic nuclei (crossed arrow). Inflammatory cell infiltration (I), and congested vasa recta (curved arrow) are also seen. (**d**) AlCl_3_/MV-treated group sections show normal shaped medulla containing loops of Henle (H) and collecting ducts (CD) while some cells are still vacuolated (arrow), and few cells have apoptotic dark nuclei (crossed arrow).

The subgroup Ia was chosen as the control group since no variations between the control subgroups could be seen. The normal architecture of the renal cortex was visible in sections taken from the control rats showing both renal corpuscles and renal tubules. Each renal corpuscle had a glomerulus (tuft of capillaries) that was encircled by Bowman’s capsule that was formed of visceral and parietal layers separated by Bowman’s space. Proximal and distal convoluted tubules make up most of the cortical renal tubules and lined with simple cuboidal epithelium with rounded nuclei with wider lumens of the distal convoluted tubules (Fig. 2a). The renal medulla composed of loops of Henle (lined with simple squamous epithelium), collecting ducts (lined with cuboidal cells having rounded nuclei), and vasa recta in between the duct system (Fig. 3a).

Upon closer examination, the AlCl_3_-treated rats’ kidney sections showed serious histopathological damage (Figs. 2b,c, 3b), like congested glomeruli surrounded by narrow Bowman’s space, extensive interstitial hemorrhage, and inflammatory cell infiltration. The chronic lesion led to congested blood vessels with thickened walls with extensive collagen deposition. The severely affected sections showed desquamated cells in the tubular lumen. Most of the tubular epithelium had small dark apoptotic nuclei with dark cytoplasm. The renal medulla also showed markedly dilated loops of Henle and collecting ducts with desquamated cells in their lumen. Inflammatory cell infiltration and clogged, dilated vasa recta were visible in the interstitial tissue.

Sections from the group treated with AlCl_3_/ASC revealed partial improvement in the histological structure. While some tubules had flattened cells with dark nuclei and cytoplasm and destroyed lumen. Others displayed normal tubular cells with acidophilic cytoplasm and vesicular nuclei. There was still evidence of interstitial bleeding. Collecting duct cells showed apoptotic nuclei, some of them seemed vacuolated. There was still evidence of inflammatory cell infiltration and clogged vasa recta (Figs. 2d,e, 3c).

AlCl_3_/MV-treated group (Figs. 2f, 3d) showed ameliorated histological structure in the form of renal corpuscles and tubules with normal shapes. Most of the tubular lining epithelium exhibited acidophilic cytoplasm and vesicular nuclei. Cortical blood vessels were dilated and congested, and interstitial hemorrhage was still present. Few of the cells with apoptotic nuclei and several vacuolated medullary tubular lining cells were present.

#### Sections stained with periodic acid Schiff reaction (PAS)

**Figure 4:**
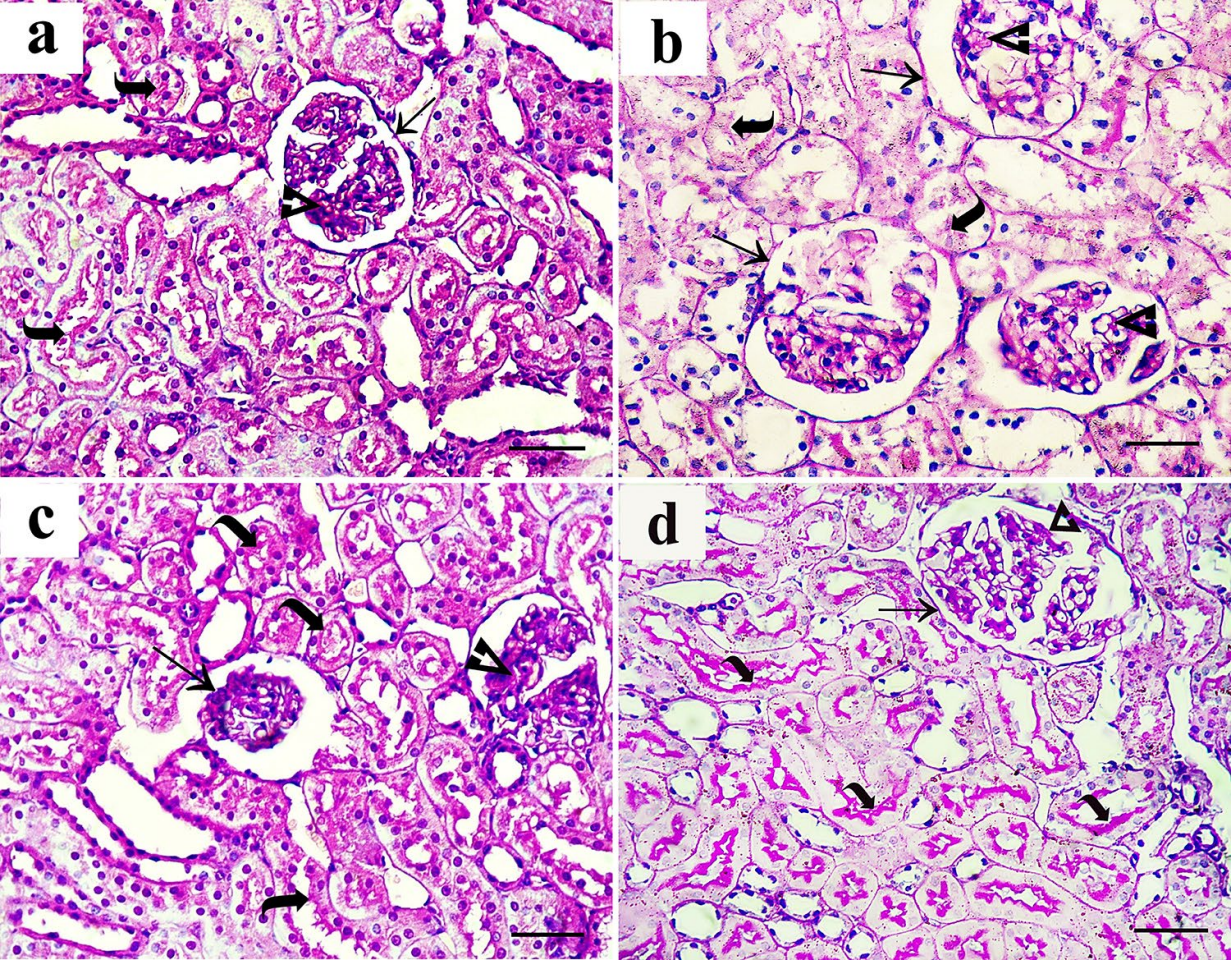
PAS-stained sections of the renal cortex show (**a**) the control group shows a strong positive reaction at glomerular capillaries (arrowhead), renal corpuscles (arrow) and basement membranes and the brush border of the tubular cells (curved arrow). (**b**) AlCl_3_-treated group shows weak PAS reaction at glomerular capillaries (arrowhead) and renal corpuscles (arrow). Complete loss of the brush border of most tubular cells (curved arrow). (**c**) The AlCl_3_/MSC-treated group shows moderate reaction in glomerular capillaries (arrowhead), renal corpuscles (arrow), and in the brush border of some tubular cells (curved arrow). (**d**) The AlCl_3_/MV-treated group shows a strong positive reaction in glomerular capillaries (arrowhead), renal corpuscles (arrow), and the brush border of most tubular cells (curved arrow).

The brush border of the tubular cells in sections of the control group’s renal cortex displayed a robust positive reaction. Additionally, glomerular capillaries, renal corpuscles, and tubule basement membranes displayed a positive response (Fig. 4a). A weak PAS reaction was visible in sections of the AlCl_3_-treated group along the brush border of most proximal convoluted tubules (PCT) which has completely vanished in several tubules. In several glomeruli, a mild positive reaction was found (Fig. 4b). The tubules in the AlCl_3_/ASC-treated group showed varying degrees of positive reaction near the brush border, with some tubules exhibiting moderate positive reaction (Fig. 4c). A robust positive reaction was found in the glomeruli, brush border, and basement membranes of many proximal convoluted tubules in the AlCl_3_/MV-treated group (Fig. 4d).

#### Mallory trichrome-stained sections

**Figure 5:**
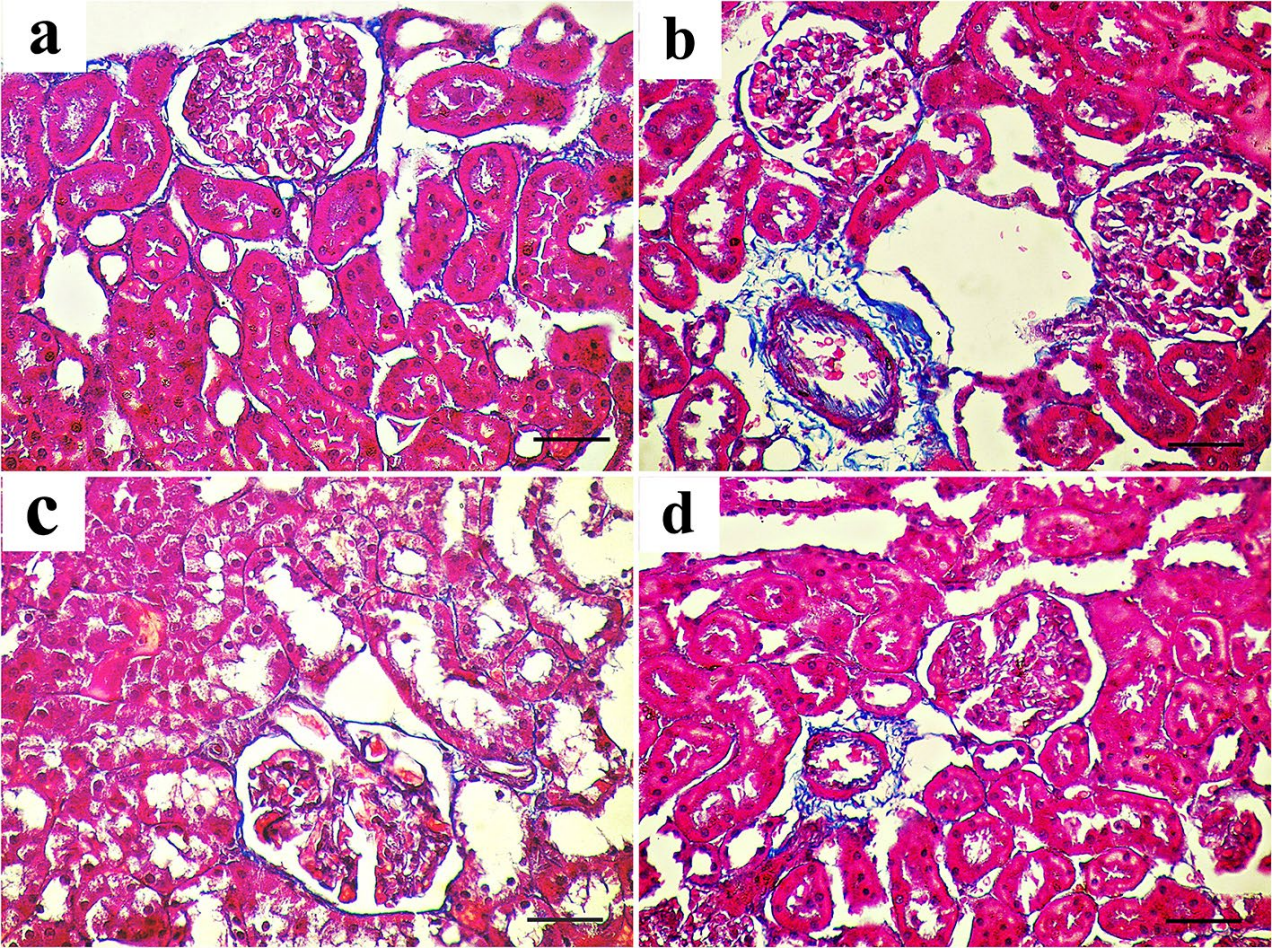
Mallory trichrome-stained sections of the renal cortex show (**a**) the control group shows few collagen fibers around the renal corpuscle (arrow) and in the interstitium between renal tubules (curved arrow). (**b**) The AlCl_3_-treated group shows excess collagen deposition around the renal corpuscle (arrow), in the interstitium between renal tubules (curved arrow), and around the blood vessels (crossed arrow). (**c,d**) The AlCl_3_/MSC-treated group and AlCl_3_/MV-treated group show a moderate amount of collagen around the renal corpuscle (arrow), in the interstitium between renal tubules (curved arrow), and around the blood vessels (crossed arrow) respectively.

In the control group, only a few collagen fibers were visible in the interstitium between the renal tubules and surrounding the renal corpuscles in sections of the renal cortex (Fig. 5a). Collagen fiber deposition in the interstitium between renal tubules and around blood vessels was higher in the AlCl_3_-treated group (Fig. 5b). Few collagen fibers were seen in the interstitium between renal tubules in the AlCl_3_/ASC-treated group (Fig. 5c). The AlCl_3_/MV-treated group’s (IV) response was essentially like that of the control group (Fig. 5d).

#### Immunohistochemical results

**Figure 6:**
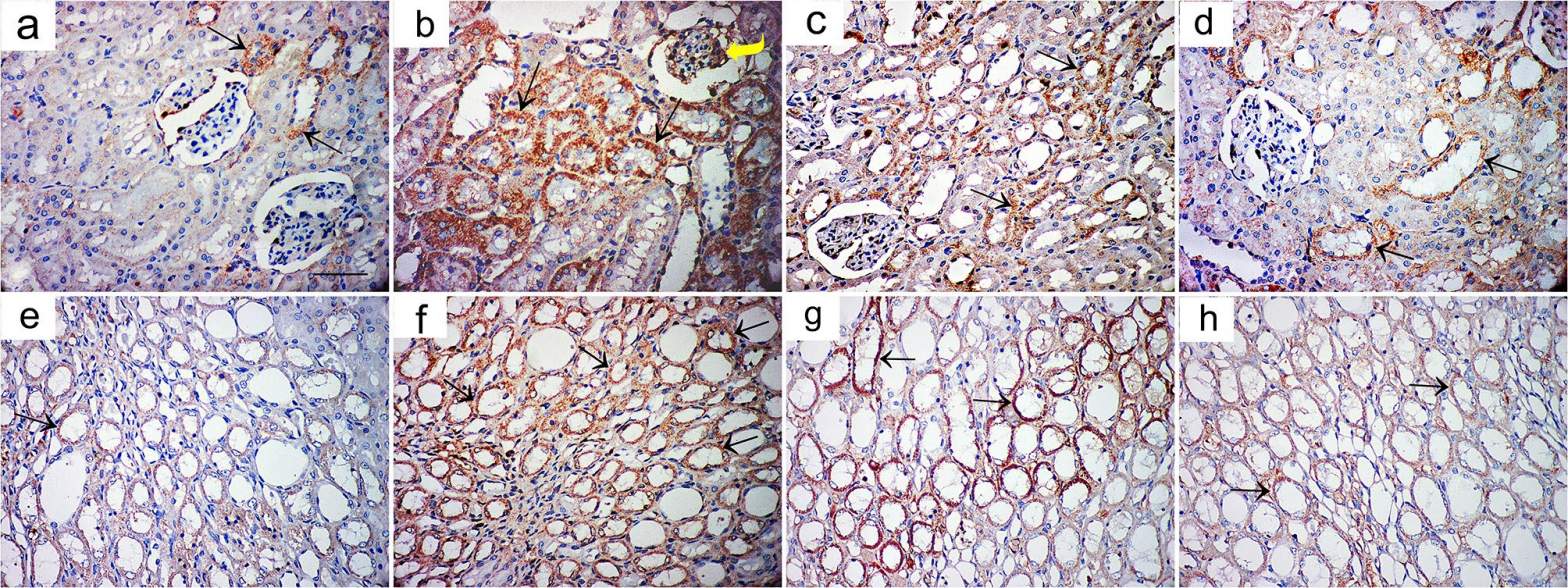
Sections stained with anti-caspase3 antibody showed minimal cytoplasmic expression in tubular epithelial cells of the cortex and few collecting ducts in the medulla of the control group (**a,e**). The AlCl_3_-treated group showed extensive expression in glomeruli, tubular cells, and medullary collecting ducts (**b,f**). The AlCl3/ASC-treated group showed positive expression in some tubular cells in both cortex and medulla (**c,g**), while the AlCl3/MV-treated group showed anti-caspase3 expression in a few cells in both cortex and medulla (**d,h**).

**Figure 7:**
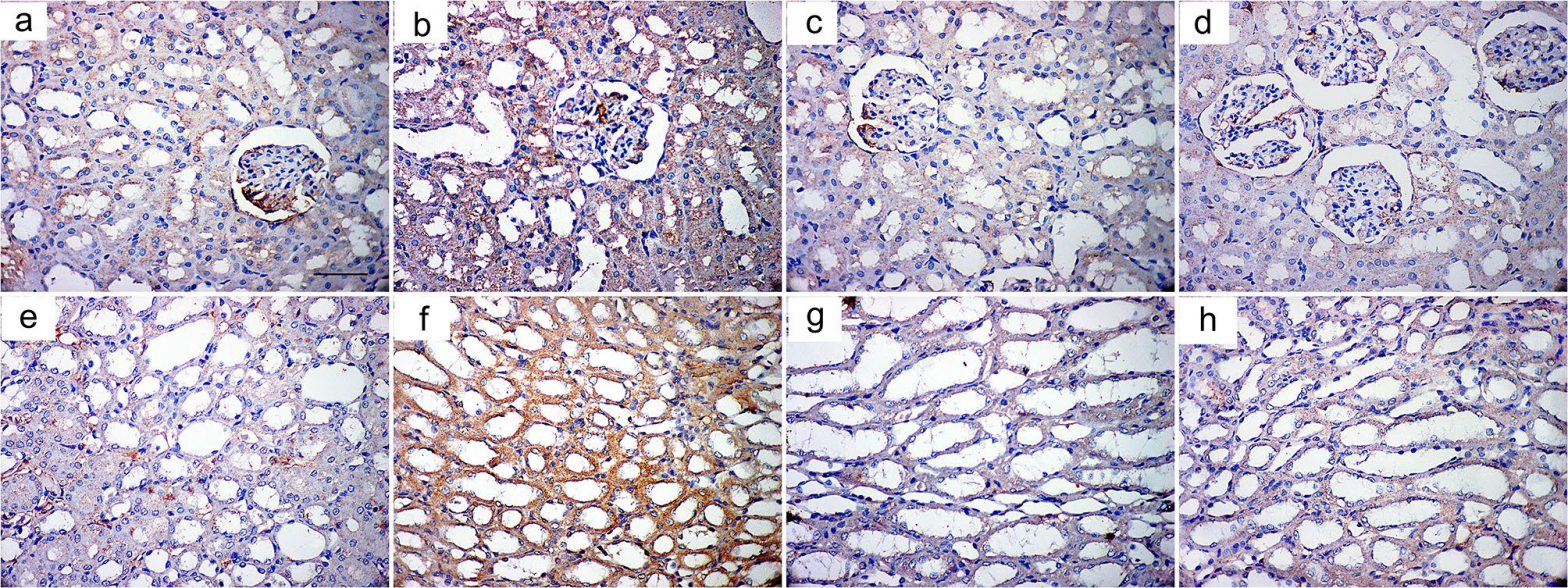
Sections stained with anti-IL1B antibody showed minimal cytoplasmic expression in both cortex and medulla of the control group (**a,e**), while strong positive reaction was detected in glomeruli, tubular cells, and medullary collecting ducts of AlCl_3_-treated group (**b,f**). Both of AlCl_3_/ASC-treated group and the AlCl_3_/MV-treated group showed weak expression in a few cells in both the cortex and medulla (**c,d,g,h** respectively).

**Figure 8:**
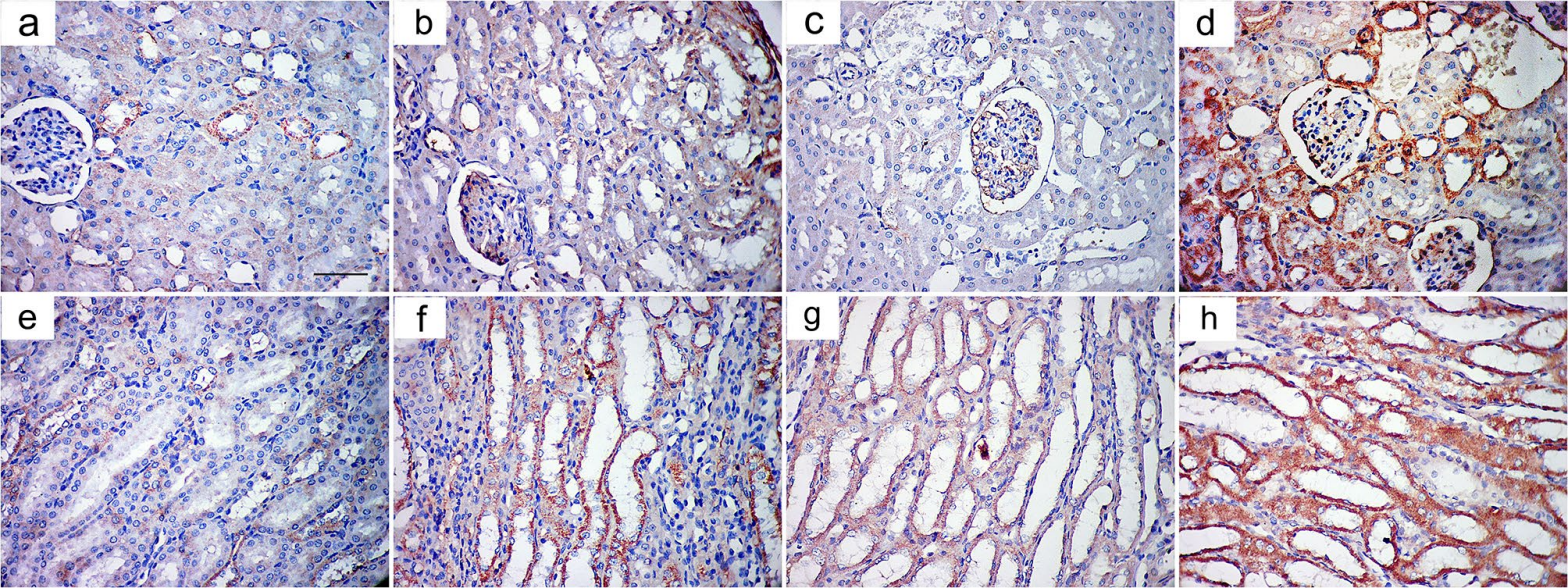
Sections stained with anti-CD40 antibody showed a very weak reaction in control group (**a,e**), and the AlCl_3_/ASC-treated group (**c,g**). AlCl_3_-treated group (**b,f**) showed moderate expression of CD40 while a strong positive reaction of CD40 was observed in AlCl_3_/MV-treated group (**d,h**).

In tubular epithelial cells of the cortex and a few collecting ducts in the medulla of the control group, sections stained with anti-caspase3 antibody revealed minimal cytoplasmic expression (Fig. 6a,e). The AlCl_3_-treated group showed extensive expression in glomeruli, tubular cells, and medullary collecting ducts (Fig. 6b,f). The AlCl_3_/ASC-treated group showed positive expression in some tubular cells in both cortex and medulla (Fig. 6c,g), while the AlCl_3_/MV-treated group showed anti-caspase3 expression in few cells in both cortex and medulla (Fig. 6d,h). Sections stained with anti-IL1B antibody showed minimal cytoplasmic expression in both the cortex and medulla of the control group (Fig. 7a,e), while in the AlCl_3_-treated group, a strong positive reaction was detected in the glomeruli, tubular cells, and medullary collecting ducts (Fig. 7b,f). AlCl_3_/ASC and AlCl_3_/MV treatment groups both revealed weak expression in a small number of cells in the cortex and medulla (Fig. 7c,d,g,h respectively). Sections stained with anti-CD40 antibody showed very weak reaction in the control group (Fig. 8a,e), and the AlCl_3_/ASC-treated group (Fig. 8c,g). CD40 expression was low in the AlCl_3_-treated group (Fig. 8b,f), whereas it was strongly positive in the AlCl_3_/MV-treated group (Fig. 8d,h).

#### Electron microscope results

**Figure 9:**
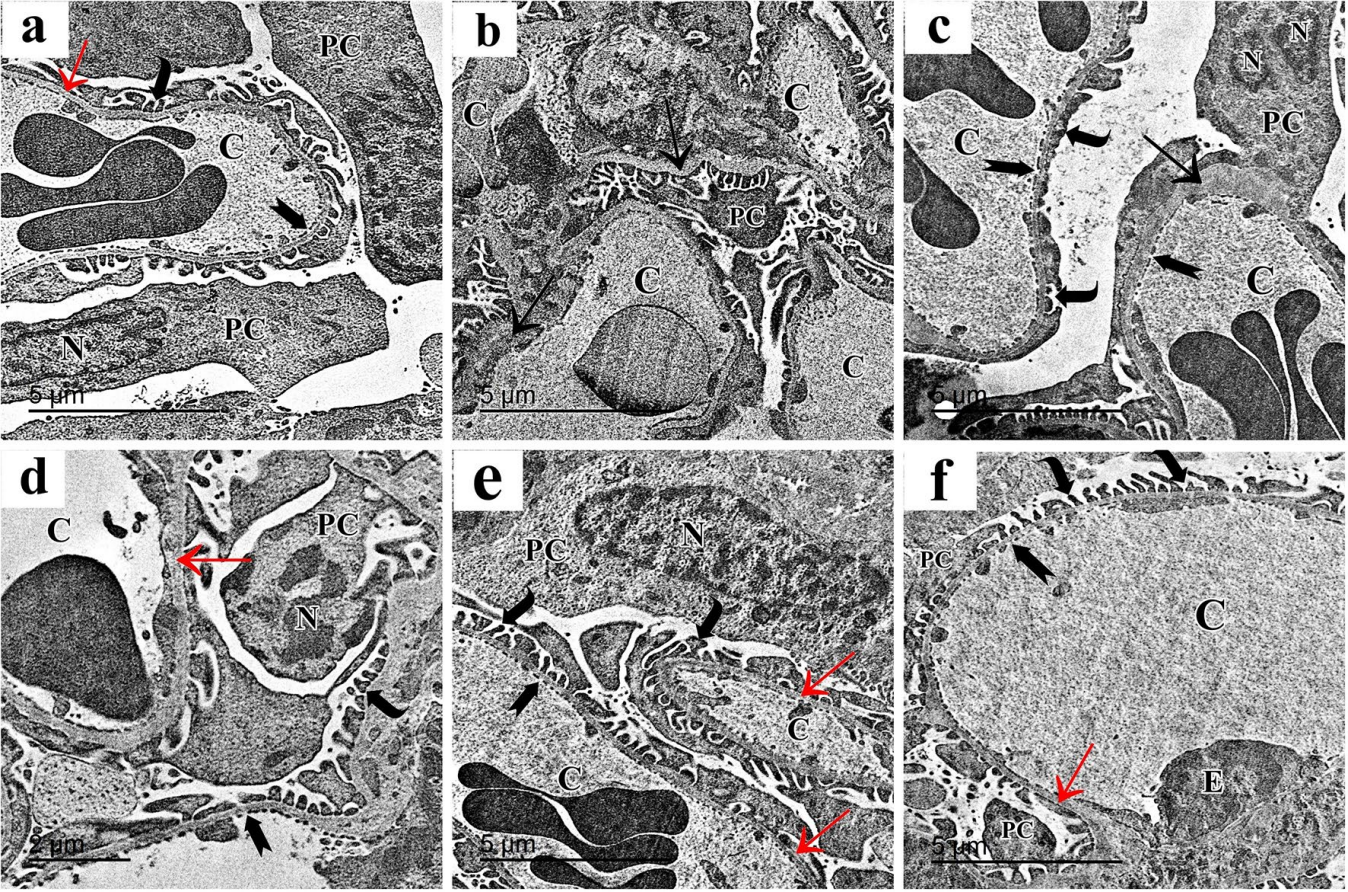
Electron micrographs of rats’ renal cortex show (**a**) the control group shows two podocytes (PC) with irregular euchromatic nuclei (N), they have primary processes (arrow), and interdigitating secondary processes (curved arrow). Glomerular capillaries (C) are lined by thin fenestrated endothelium (bifid arrow). The glomerular basement membrane (red arrow) is uniform, and thin and appears tri laminar. (**b**) The AlCl_3_-treated group shows part of a podocyte’s primary process (PC) in between congested capillaries (C). The glomerular basement membrane (arrow) is thick and irregular. (**c**) Another section of the same group shows the heterochromatic nucleus (N) of the podocyte (PC) with effacement of the podocyte foot processes (curved arrow). Congested glomerular capillaries (C) lined by fenestrated thin endothelium (bifid arrow). The glomerular basement membrane (arrow) is thickened. (**d**) AlCl_3_/MSC-treated group shows irregular heterochromatic nucleus (N) of the podocyte (PC), normal appearance of foot processes (curved arrow), fenestrated blood capillary endothelium (bifid arrow), and thickened glomerular basement membrane (red arrow). (**e,f**) AlCl_3_/MV-treated group shows elongated nucleus (N) of podocyte (PC) with clumps of heterochromatin and normal foot processes (curved arrow). Thin fenestrated (bifid arrow) blood capillary endothelium (E), and thin uniform glomerular basement membrane (red arrow) can be seen.

Ultrathin sections obtained from the renal cortex of the control group exhibit normal ultrastructure. Podocytes contained irregular euchromatic nuclei with primary and interdigitating secondary processes, demonstrating normal ultrastructure. Thin fenestrated endothelium bordered the glomerular capillaries. The glomerular basement membrane was homogeneous, thin, and seemed trilaminar (Fig. 9a). The AlCl_3_-treated group showed thick and irregular glomerular basement membrane and heterochromatic nuclei of the podocytes with effacement of the podocyte foot processes. Glomerular capillaries were lined by fenestrated thin endothelium and appeared congested (Fig. 9b,c). The group that was exposed to AlCl_3_/MSC exhibited thickened glomerular basement membrane and irregular heterochromatic nuclei of the podocytes but normal appearance of foot processes (Fig. 9d). AlCl_3_/MV-treated group showed normal podocyte nucleus and foot processes. Thin fenestrated blood capillary endothelium and thin uniform glomerular basement membrane (Fig. 9e,f).

**Figure 10:**
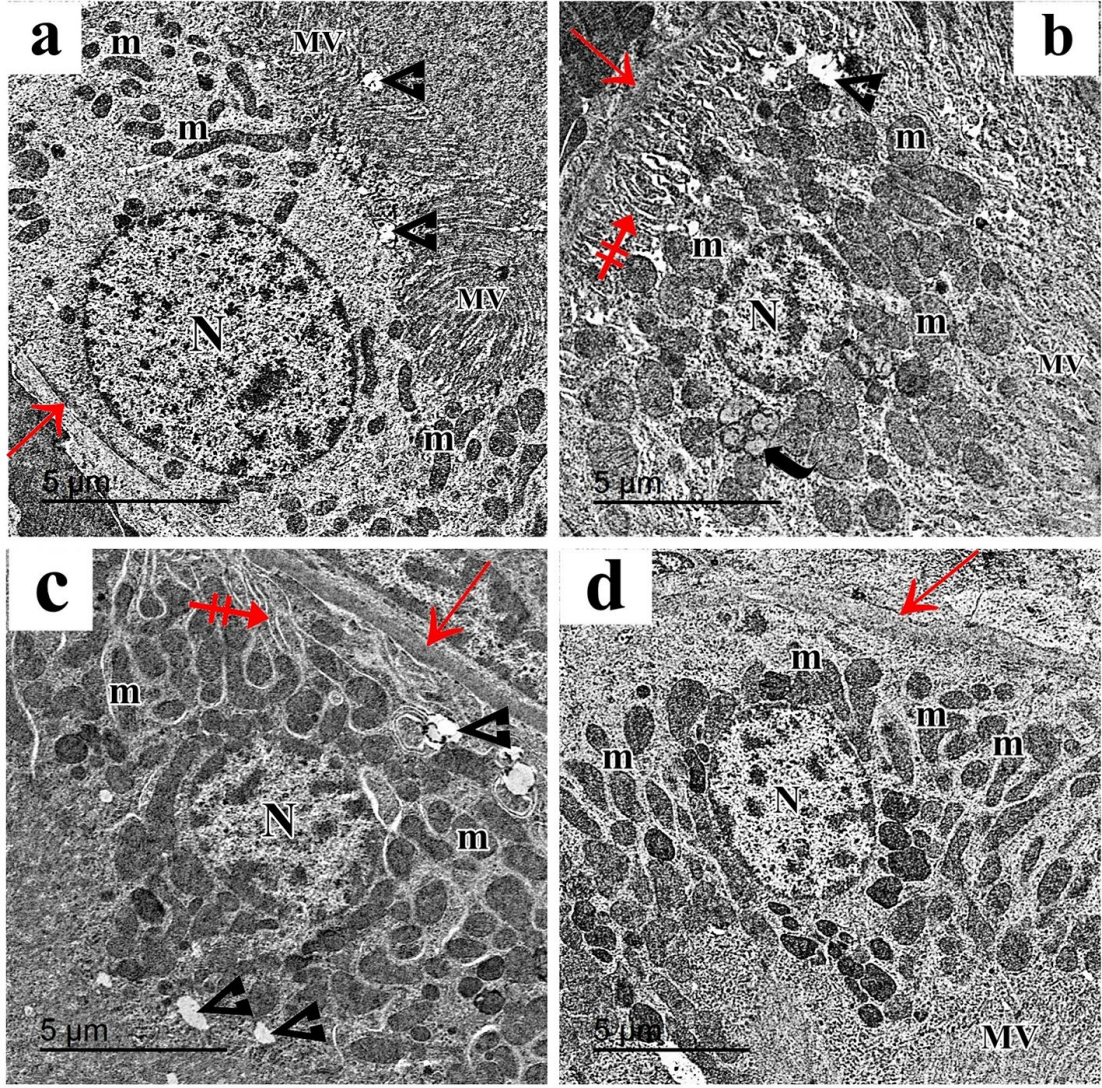
Electron micrographs of the PCT show (**a**) the control group shows the tubular epithelial cells have rounded euchromatic nucleus (N), numerous elongated mitochondria (m), apical closely packed microvilli (MV) and apical small pinocytotic vesicles (arrowhead). A thin basement membrane is noticed (red arrow). (**b**) The AlCl_3_-treated group shows PCT cells with a shrunken heterochromatic nucleus (N), swollen mitochondria (m), cytoplasmic vacuolation (arrowhead), and multivesicular body (curved arrow) can be seen. The apical compartment shows distorted microvilli (MV), and the basal compartment shows a thickened basement membrane (red arrow) and attenuated basal infoldings (crossed arrow) empty of mitochondria. (**c**) AlCl_3_/MSC-treated group shows PCT with heterochromatic nucleus (N), numerous elongated and rounded mitochondria (m), cytoplasmic vacuolation (arrowhead), thickened basement membrane (red arrow) and basal infoldings (crossed arrow) containing mitochondria. (**d**) PCT of the AlCl_3_/MV-treated group shows a rounded euchromatic nucleus (N) surrounded by numerous mitochondria (m). Apical closely packed microvilli (MV) and a thin basement membrane are noticed (red arrow).

Ultrathin sections of the control group proximal convoluted tubules (PCT) showed tubular cells with rounded euchromatic nuclei, numerous elongated mitochondria, apical tiny pinocytotic vesicles, and densely packed microvilli. Cells rested on a thin basement membrane (Fig. 10a). In the AlCl_3_-treated group, PCT cells showed shrunken heterochromatic nuclei, swollen mitochondria, and cytoplasmic vacuolations. The apical compartment showed distorted microvilli, and the basal compartment showed thickened basement membrane and attenuated basal infoldings (Fig. 10b). In AlCl_3_/MSC-treated group, PCT still had heterochromatic nuclei, cytoplasmic vacuolations and thickened basement membrane (Fig. 10c). PCT of AlCl_3_/MV-treated group had rounded euchromatic nuclei surrounded by numerous mitochondria, apical closely packed microvilli, and a thin basement membrane (Fig. 10d).

**Figure 11:**
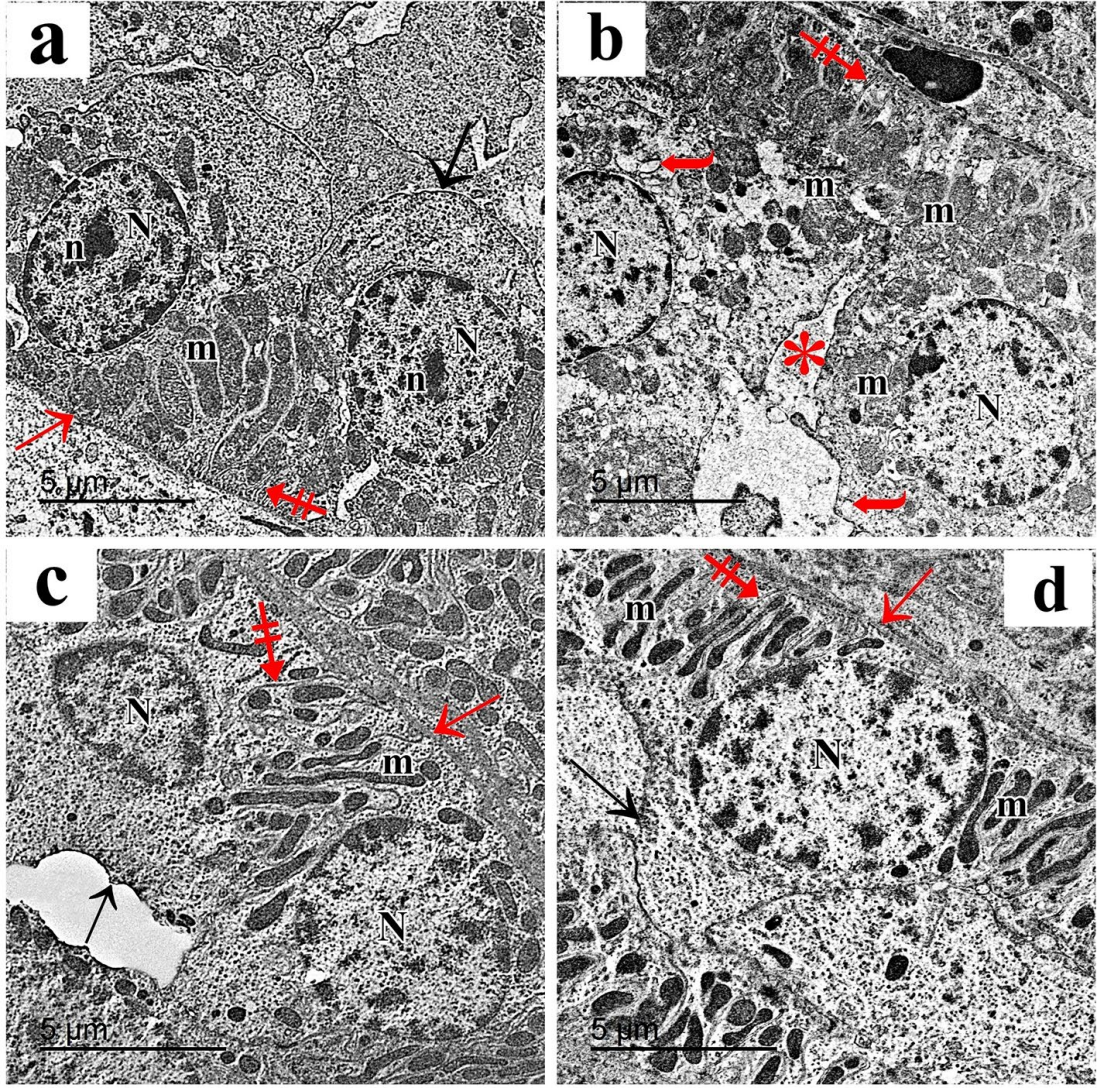
Electron micrographs of the DCT show (**a**) The control group shows the tubular epithelial cells have apical smooth cell membrane (arrow), rounded euchromatic nuclei (N) with prominent nucleoli (n), numerous elongated basal mitochondria (m) located within the basal infoldings (crossed arrow). A thin basement membrane is also seen (red arrow). (**b**) The AlCl_3_-treated group shows DCT cells with rounded nuclei (N), few rounded basal mitochondria (m), attenuated basal infoldings (crossed arrow), and cytoplasmic vacuolations (curved arrow). Wide intercellular spaces can be seen (asterisk). (**c**) AlCl_3_/MSC-treated group shows DCT epithelium with heterochromatic nuclei (N), elongated basal mitochondria (m) located within the basal infoldings (crossed arrow) and resting on a thin basement membrane (red arrow). The irregular apical cell membrane can be seen (arrow). (**d**) DCT of the AlCl_3_/MV-treated group shows normal tubular epithelial cells with the apical smooth cell membrane (arrow), rounded euchromatic nuclei (N), numerous basal elongated mitochondria (m) located within the basal infoldings (crossed arrow) and resting on a thin basement membrane (red arrow).

The tubular epithelial cells in the control c (DCT) were ultrastructurally examined and revealed to have multiple elongated basal mitochondria inside of the basal infoldings, rounded euchromatic nuclei, and smooth apical cell membranes. Cells were supported by a thin basement membrane (Fig. 11a). AlCl_3_ treatment markedly affected the DCT cells. They showed cytoplasmic vacuolations and few rounded basal mitochondria in attenuated basal infoldings. Cells were separated with wide intercellular spaces (Fig. 11b). The AlCl_3_/ASC-treated group showed DCT epithelium with heterochromatic nuclei, elongated basal mitochondria located within the basal infoldings and resting on a thin basement membrane (Fig. 11c). AlCl_3_/MV-treated group’s DCT revealed typical tubular epithelial cells with an apical smooth cell membrane, rounded euchromatic nuclei, and many basal elongated mitochondria found within the basal infoldings and resting on a thin basement membrane. (Fig. 11d).

### Morphometric and statistical results

**Figure 12:**
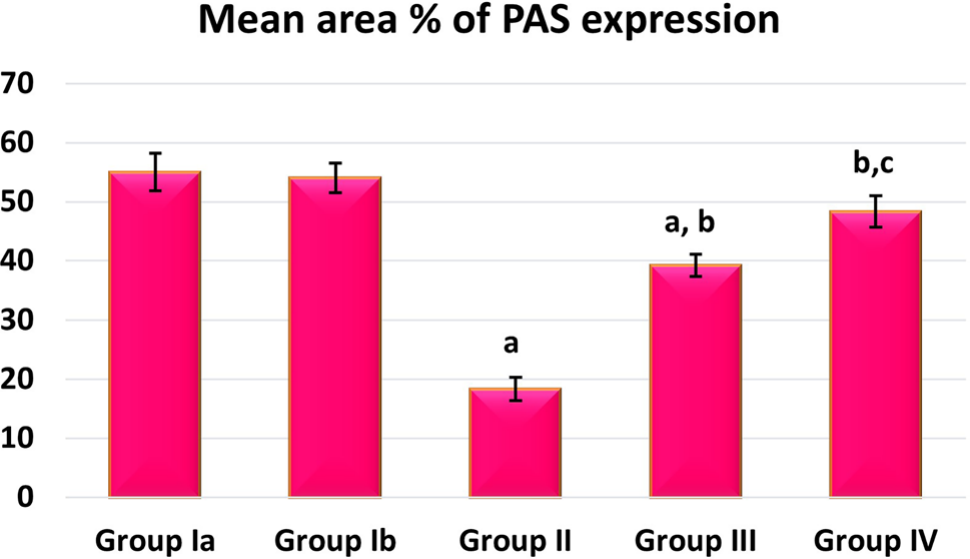
Mean area % of PAS reaction in studied groups. (**a**) Significant with control group I, (**b**) significant with group II, (**c**) significant with group III.

**Figure 13:**
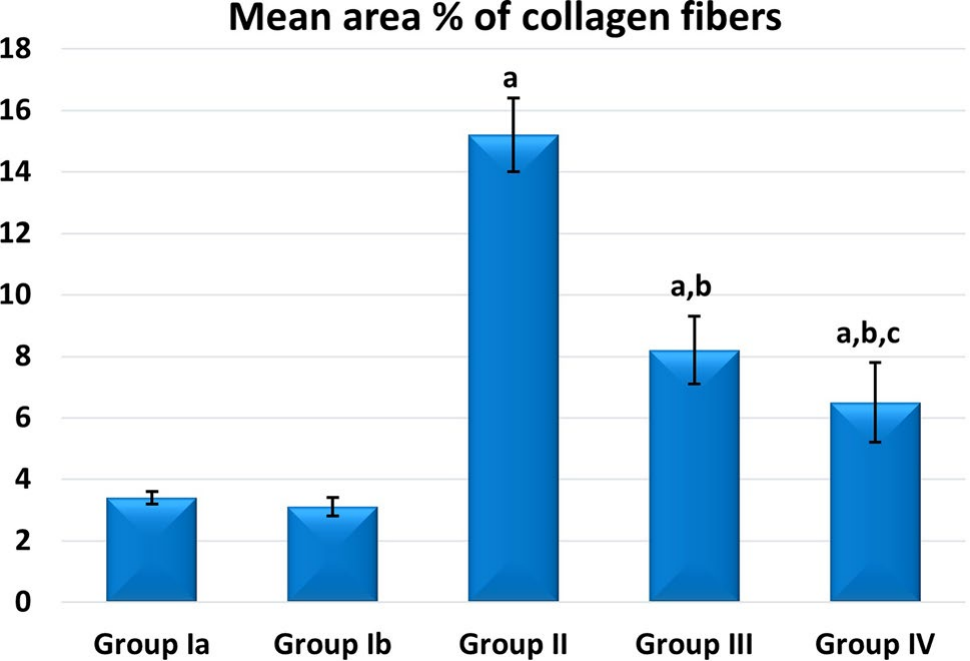
Mean area % of collagen fibers deposition in studied groups. (**a**) Significant with control group I, (**b**) significant with group II, (**c**) significant with group III.

**Figure 14:**
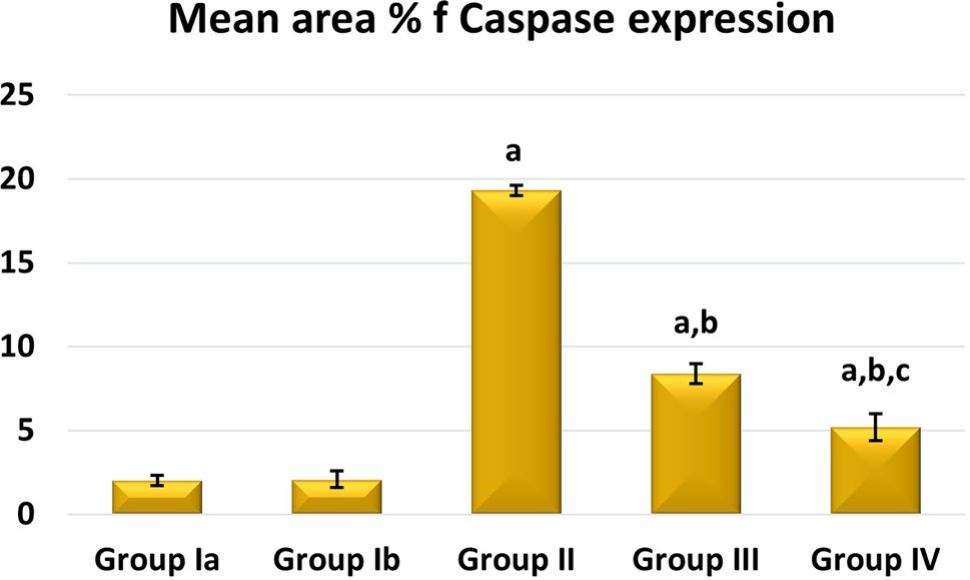
Mean area % of Caspase3 immunoexpression in studied groups. (**a**) Significant with control group I, (**b**) significant with group II, (**c**) significant with group III.

A high statistically significant decrease in the mean area % of PAS expression was noticed in groups II and III relative to control with the least values observed in group II. Groups III and IV showed significant improvement compared to group II while better PAS expression was noticed with group IV (Fig. 12). Regarding the mean area % of collagen fiber deposition, a highly significant increase was noticed in groups II, III, and IV relative to control with the highest levels noticed in group II. Groups III and IV showed significant improvement to Group II, and the lowest expression levels were observed in Group IV (Fig. 13). Regarding apoptosis expression, a highly significant increase in the mean area % of caspase3 immunoexpression was noticed in Groups II and III, and IV relative to control with the highest levels noticed in group II. Groups III and IV showed a significant decrease in apoptosis compared to Group II, and the lowest expression levels were observed in Group IV (Fig. 14).

**Figure 15:**
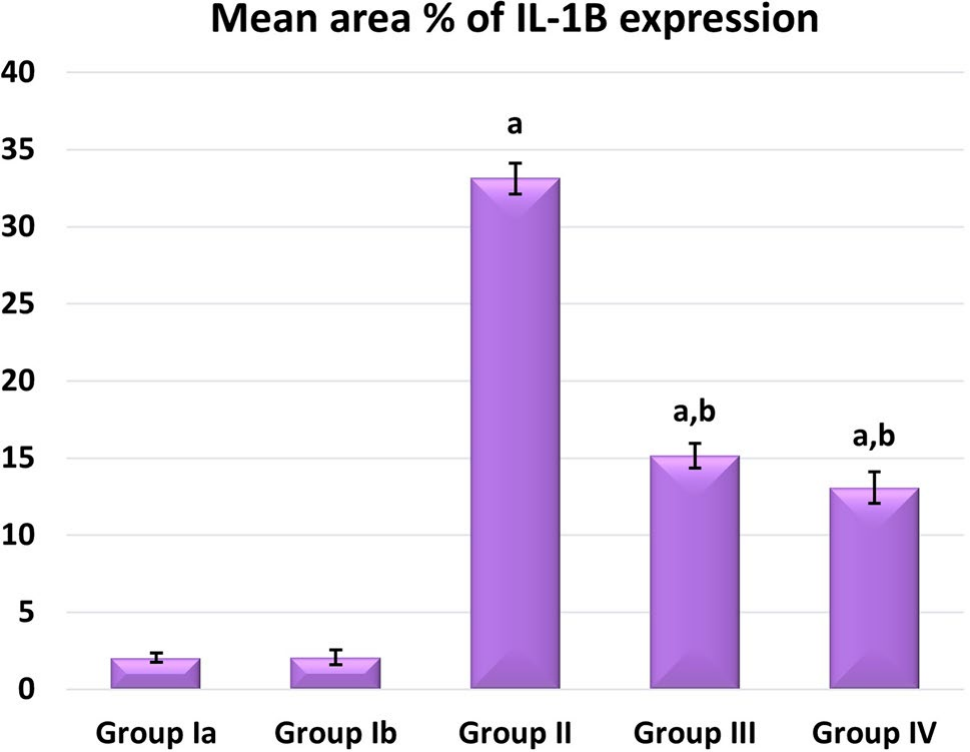
Mean area % of IL1B immunoexpression in studied groups. (**a**) Significant with control group I, (**b**) significant with group II, (**c**) significant with group III.

**Figure 16:**
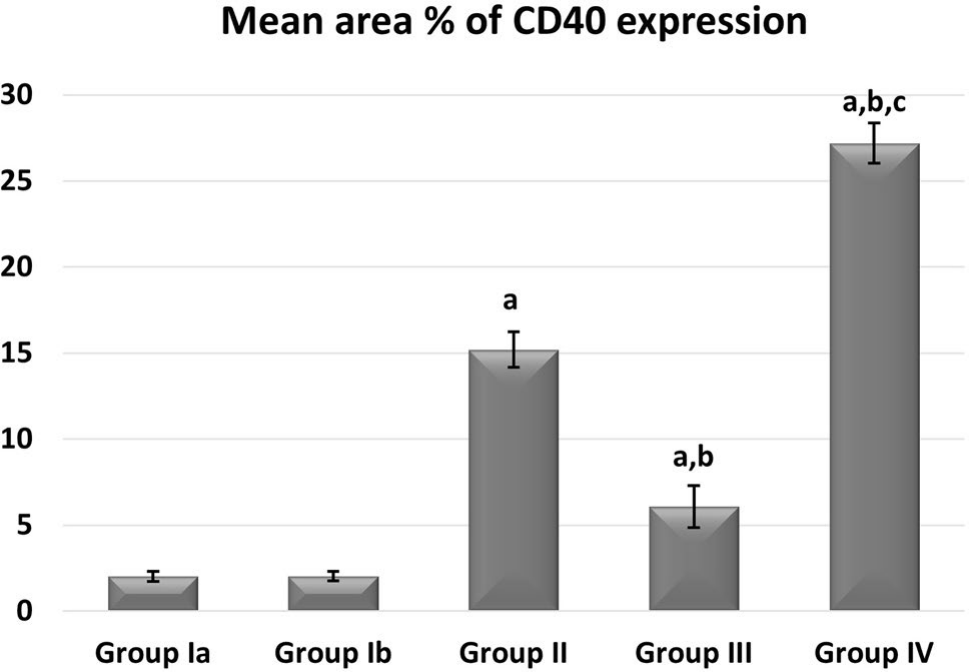
Mean area % of CD40 immunoexpression reaction in studied groups. (**a**) Significant with control group I, (**b**) significant with group II, (**c**) significant with group III.

Regarding inflammatory cytokine IL1B expression, the highest levels of its expression were noticed in group II. Groups III and IV significantly reduced their expression compared to Group II, with no changes between the two groups being noticed (Fig. 15). Regarding immunoexpression of macrovesicles’ marker CD40, groups II, III, and IV were significantly increased than control. Group III showed lower expression levels than Group II while the highest expression levels were only noticed in Group IV (Fig. 16).

## Discussion

The current study was conducted to compare the therapeutic potential of ASC versus MVs in ameliorating AlCl_3_-induced chronic renal damage by investigating several biochemical and histological parameters. Most of the published research that examined the toxic effects of AlCl_3_ in animals used parenteral or intraperitoneal routes, which don’t represent the primary route of exposure for humans. Oral ingestion has the highest toxicological effects even though only a small amount of Al is absorbed through the digestive tract^63,64^. Therefore, to simulate the long-term Al toxicity, rats in the present study were given AlCl_3_ through an intragastric tube for 40 days. Because the intestine acts as a protective barrier against Al toxicity and only a small portion (0.1–0.5%) of ingested Al is absorbed, a high dose of oral AlCl_3_ was chosen^65^.

Chronic ingestion of AlCl_3_ disturbed the antioxidant capacity of the renal tissue which led to glomerular and tubular ultrastructural changes, resulting in deteriorated kidney function. According to Ref.^66^, the toxic effect of Al is mediated by cell membrane damage in which Al binds to the ferric iron-carrying protein transferrin, so it reduces the binding of ferrous iron. Increased intracellular ferrous iron levels cause lipid membrane peroxidation, increased membrane permeability, and receptor function modification^5^. This mechanism explains our study’s elevated renal tissue MDA level due to membrane peroxidation.

Moreover, the decreased expression levels of the antioxidant parameters (SOD, GSH, and TAC) agreed with the findings of Ref.^67^. They stated that the excretion of numerous xenobiotics, pollutants, and poisons by the kidneys makes them vulnerable to the release of large amounts of free radicals, which raises oxidative stress and plays a role in the pathogenesis of kidney disease. Sargazi et al*.*^68^ demonstrated that AlCl_3_’s harmful effects were secondary to the production of ROS, which in turn caused oxidative degradation of cellular lipids, proteins, and DNA. Rats chronically exposed to AlCl_3_ developed nephrotoxicity according to Stacchiotti et al*.*^69^. Reduced glutathione inside cells was decreased in Al intoxication. Al salts may also stop the activity of several enzymes, including phosphodiesterase and acid and alkaline phosphatases^70,71^. Critical accumulation of Al in the kidneys can result in elevated serum urea and creatinine levels, which can ultimately lead to renal failure according to Refs.^72,73^.

AlCl_3_ chronic exposure also exaggerated the levels of the pro-inflammatory and lowered the anti-inflammatory cytokines in serum and renal tissue as detected biochemically and by immunohistochemical study. Our findings agreed with those of Refs.^74,75^. Moreover, Refs.^76,77^ proved that rats administrated AlCl_3_ exhibited a significant increase in the brain IL-2, IL-6, and TNF-α expression levels compared to the control group. Likewise, Ref.^78^ reported that a concentration of 53.5 mg/L dose of AlCl_3_ added to the drinking water had significantly elevated the serum inflammatory markers IL-6 and TNF-α. Accumulation of Al was also accompanied by the release of mitochondrial cytochrome c, which eventually causes increased production of free radicals and increased oxidative damage with exaggeration of proinflammatory cytokines^79^.

The damaging effect of AlCl_3_ was also noticed in both the renal cortex and medulla in the form of congested glomeruli, extensive interstitial hemorrhage, desquamated cells in the tubular lumen, and tubular necrosis. It influenced the Bowman’s capsule by thickening the basement membrane, which filters blood to make urine. Additionally, it resulted in damaged proximal and distal tubules^39,68,69^. These degenerative changes resulted from the production of reactive oxygen species (ROS) with oxidative damage to cellular lipids, proteins, and DNAs^5,80^. The membrane-damaging effect of AlCl_3_ was proved by PAS examination of renal tubules. The brush border completely vanished from some tubules.

A well-expected outcome of the previous findings is the increased collagen fiber deposition in the interstitium between renal tubules in the AlCl_3_ group. The crosstalk between the inflammatory cells and fibroblasts resulted in more fibrosis. Saad et al*.*^5^ acquired the same data and found that AlCl_3_ significantly induced interstitial fibrosis and marked cellular invasion with inflammatory cells. Macrophages are involved in inflammatory reactions with the expression of many pro-inflammatory cytokines, such as TNF-α, IL-1β, IL-6, and IL-12 primarily through the production of reactive oxygen and nitrogen species^81^. Moreover, Jian et al*.*^82^ stated that macrophage subsets can produce large amounts of pro-fibrotic growth factors, such as IL-10, IGF-1, platelet-derived growth factors, and fibroblast growth factors, which explain enhanced fibrosis in our study. Witherel et al*.*^83^ explained that macrophage–fibroblast crosstalk enhances fibrosis, as macrophages secret inflammatory cytokines such as IL1-B, IL-6, TNFα, and TGFB which stimulate fibroblast proliferation and transformation into myofibroblasts with more collagen fibers deposition.

We used ASCs by intravenous route as a new strategy in the treatment of metal-induced chronic renal toxicity. Although vein transplantation cannot be selectively distributed to the kidney in large quantities, the main role of exogenous MSCs is to enhance the endogenous stem cells of the kidney to play a role according to Humphreys and Bonventre^84^. In acute kidney injury (AKI) models, most of the regenerated cells were derived from renal cells, and the role of MSCs was to enhance the proliferation of these cells. Therefore, the degree of renal regeneration was limited by the regenerative potential of its endogenous cells, with no relation to the number of transplanted MSCs^84,85^. MSC therapy recovers renal function through various mechanisms such as anti-oxidative stress, anti-inflammation, anti-apoptosis, and angiogenesis. They promote angiogenesis through paracrine secretion of bioactive substances such as VEGF, hypoxia-inducible factor 1-alpha (HIF-1α), platelet-derived growth factor-BB (PDGF-BB), and stromal cell-derived factor 1 (SDF-1)^86,87^.

In terms of safety, potency, and effectiveness, MSCs-derived microvesicles (MVs) represent an alternative therapeutic approach in regenerative medicine to avoid the complications of using stem cells such as malignancies^88^. Treatment with ASCs-derived MVs in the present study resulted in the restoration of the normal histological appearance of both the renal cortex and medulla. Our results confirmed their antioxidant, antifibrotic, antiapoptotic, and anti-inflammatory effects as reported by biochemical and histopathological examination. Recent studies also confirmed the role of MVs in treating kidney diseases^89^. For example, it slowed the progression of ischemic–reperfusion injury (IRI) by lowering the expression levels of inflammatory factors IL-6, TNF-α, NF-κB, and IFN-γ^90^. Gao et al*.*^91^ showed their inhibitory effect on inflammation of sepsis-induced AKI by blocking the NF-κB pathway. They also represent a promising therapeutic approach for preventing CKD progression by reducing inflammation and degeneration^92^. Moreover, Song et al*.*^93^ stated that extracellular vesicles serve as effective therapeutic strategies for CKD via upregulating anti-inflammatory M2 macrophages and regulatory T-cell numbers.

Concerning their anti-apoptotic effects, MSCs-derived exosomes inhibited apoptosis of NRK-52E cells induced by cisplatin via activation of the ERK1/2 pathway according to^12,94^ have also recently illustrated their role in prohibiting CKD by interfering with fibrosis and apoptosis by transferring miRNAs (e.g., miR-199a-3p44 and miR-424-5p81). Moreover, exosomes released from melatonin-preconditioned MSCs blocked apoptosis by decreasing the levels of caspase-367.

Regarding their anti-fibrotic effect, Grange et al*.*^95^ found that MVs can reverse the progression of glomerular and tubule-interstitial fibrosis in the diabetic nephritis (DN) mouse models by downregulating fibrosis-related genes (Serpia1a, TIMP1, MMP3, collagen I, and Snail). Liu et al*.*^96^ also attributed their anti-fibrotic effects in CKD to the inactivation of the reactive oxygen species (ROS)—mediated p38 mitogen-activated protein kinases/extracellular signal-regulated kinase (MAPK/ERK) pathway. Cao et al*.*^97^ added that human placenta MSCs-Exosomes reduced oxidative stress and mitochondrial fragmentation in a renal IRI model via activation of the Nrf2/keap1 pathway. In the study of Alasmari et al*.*^92^, they proved the capability of MSCs-Exo to reduce the expression of IL-6, ICAM-1, and TNF-α thus reducing fibrotic lesions. The effects of exosomes were more powerful in ameliorating kidney damage compared to MSCs. In addition, they restored kidney antioxidative defense (SOD, GPx, and CAT), reduced kidney lipid peroxidation (MDA), and reduced IL-6 and COX2 expression. Their role in suppressing renal oxidative stress and inflammation could explain tissue repair and ameliorate kidney function^98^.

In addition to the previously discussed mechanisms, MVs’ activity also involves the horizontal transfer of genetic materials, such as mRNA and miRNA, from the cell of origin to the recipient cells. This transfer causes changes in the recipient cell’s phenotypic and behavior. This could encourage these cells to reenter the cell cycle, promoting tissue regeneration^99,100^. For example, they secrete insulin-like growth factor (IGF-1) receptor mRNA directly to renal tubular epithelial cells, as well as directly secreting IGF-1 and carrying IGF-1 receptors to promote kidney repair in AKI^91^. Several studies have confirmed that MSCs-Exo enriched with miRNAs (miR-15a, miR-15b, and miR1690) and/or chemokine receptors (CCR294 and CXCR495) could ameliorate inflammation and kidney injury by reducing chemokines CX3CL196 and CCL297^101^. Higher renal miR-150 levels were associated with a worse prognosis in minimal change disease^102^. Higher plasma miR-150 was correlated with faster renal function decline in chronic kidney disease patients^103^. Previous studies reported that miR-150 increased mainly in renal tubular cells and moderately in podocytes in the repeated renal biopsies of flared American Lupus nephritis patients. miR-150 also promoted renal fibrosis by downregulating the suppressor of cytokine signal 1 (SOCS1) through in vitro study^104^. The deletion of miR-150 alleviates renal tubulointerstitial fibrosis in mice after 8 weeks of ischemia/reperfusion (I/R) by increasing collagen type I and connective tissue growth factor^105^.

## Conclusion

ASCs-derived MVs showed a more efficient therapeutic effect on AlCl_3_-induced chronic renal injury than ASCs. It was achieved by maintaining kidney tissue antioxidant defenses and reducing inflammatory cytokines levels. Moreover, modulation of the gene expression levels of miR-150-5p and renal MALT1 resulted in normalizing kidney function. The net result was improving the histopathological alterations observed in the kidney with decreased renal tissue apoptosis, fibrosis, and inflammation.

## Data Availability

All data supporting the findings of this study are available within the paper from: https://drive.google.com/drive/folders/1o46LlJojxJOUedMWjn0mSw7oexE7w5nO?usp=drive_link.
